# Variation in the Conservation of Species-Specific Gene Sets for HMO Degradation and Its Effects on HMO Utilization in Bifidobacteria

**DOI:** 10.3390/nu16121893

**Published:** 2024-06-15

**Authors:** Gerben D. A. Hermes, Christine Rasmussen, Anja Wellejus

**Affiliations:** Human Health Research, Human Health Biosolutions, Novonesis, Kogle Alle 6, 2970 Hoersholm, Denmarkanjwe@novonesis.com (A.W.)

**Keywords:** *Bifidobacterium*, human milk oligosaccharides, probiotics, synbiotics, infant, microbiota, genomics

## Abstract

Human milk provides essential nutrients for infants but also consists of human milk oligosaccharides (HMOs), which are resistant to digestion by the infant. Bifidobacteria are among the first colonizers, providing various health benefits for the host. This is largely facilitated by their ability to efficiently metabolize HMOs in a species-specific way. Nevertheless, these abilities can vary significantly by strain, and our understanding of the mechanisms applied by different strains from the same species remains incomplete. Therefore, we assessed the effects of strain-level genomic variation in HMO utilization genes on growth on HMOs in 130 strains from 10 species of human associated bifidobacteria. Our findings highlight the extent of genetic diversity between strains of the same species and demonstrate the effects on species-specific HMO utilization, which in most species is largely retained through the conservation of a core set of genes or the presence of redundant pathways. These data will help to refine our understanding of the genetic factors that contribute to the persistence of individual strains and will provide a better mechanistic rationale for the development and optimization of new early-life microbiota-modulating products to improve infant health.

## 1. Introduction

Aberrant human gut microbiome development during infancy is linked to various health conditions that can manifest themselves throughout adulthood. The colonization of pioneer microbes shortly after birth represents a key first step in this mutualistic relationship, shaping the developing microbiota and impacting numerous host physiological processes [1]. This early-life developmental window represents a critical time for microbe–host interactions, as the foundations for future health are established here [2]. Breastfeeding is considered one of the most efficient strategies to shape a healthy gut microbiome during infancy [3]; however, its formation is also influenced by various other external factors such as the delivery mode, antibiotic use, and the environment [4,5,6,7,8,9]. Bifidobacteria are among the first colonizers and commonly represent over 70% of the total microbial community during breastfeeding, providing various health benefits for the host [10].

Bifidobacteria are Gram-positive, non-motile, non-spore-forming bacteria belonging to the phylum Actinobacteria [11]. The species *B. breve*, *B. longum* subsp. *longum* (*B. longum*)*, B. longum* subsp. *infantis* (*B. infantis*), and *B. bifidum* typically dominate the infant gut, with *B. pseudocatenulatum*, *B. longum* subsp. *suis*, *B. catenulatum*, and *B. kashiwanohense* occurring at lower frequency rates [12,13,14,15,16,17,18,19,20,21]. However, this infant–adult divide is ambiguous, considering the vertical transfer of adult bifidobacterial strains from mother to infant occurs during birth [8].

The early colonization by bifidobacteria is largely facilitated by their ability to efficiently metabolize human milk oligosaccharides (HMOs). Despite being the third most abundant solid component in breastmilk, they provide no (direct) nutritional value to the infant [22]. Instead, HMOs act as compounds that selectively promote the growth of mostly bifidobacteria; thus, the production of different HMOs has been industrialized to fortify formula milk [23].

The human milk glycans are diverse, and over 100 different HMO structures have been identified. This structural diversity is achieved with five building blocks—glucose, galactose, *N*-acetylglucosamine, fucose, and sialic acid. The core consists of lactose at the reducing end, often elongated by repeats of β1–3-linked lacto-*N*-biose (Galβ1–3GlcNAc; LNB), termed type 1 chains, or by β1–3/6-linked *N*-acetyllactosamine (Galβ1-4GlcNAc; LacNAc), termed type 2 chains. These core structures are frequently decorated with fucose and sialic acid residues via α1–2/3/4 and α2–3/6 linkages, respectively. Despite the structural diversity, a small number of HMO species can represent up to 70% of the total, and typically fucosylated HMOs make up the majority (>40%), unless the milk is derived from non-secretor or Lewis-negative mothers [24,25].

The HMO utilization profiles differ significantly between infant-gut-associated *Bifidobacterium* species. *B. bifidum* and *B. infantis* can utilize many different HMOs directly, while most species generally only metabolize a limited range of HMOs [15,17,19,20,26,27,28,29,30,31,32,33], thereby relying on cross-feeding strategies with other species [34,35]. The infant-gut-associated bifidobacteria have evolved two different strategies to decompose HMOs. In one case, the HMOs are externally hydrolyzed with cell-membrane-attached glycosyl hydrolases (GHs), and the resulting mono- and di-saccharides are taken up and further degraded internally. In the second case, the HMOs are internalized using ATP-binding cassette (ABC) transporters and degraded internally in a sequential manner by GHs. Genetic and enzymatic approaches have shown that bifidobacteria encode GHs with specificity towards all HMO linkages. However, this repertoire is species-specific, resulting in different HMO utilization abilities. The α1,2 (GH29) and α1,3/4 (GH95) fucosidases, α2,3/6 sialidases (GH33), β-galactosidases for β1,4 (GH2) and β1,3 (GH42), beta-*N*-acetylglucosaminidase (GH20), lacto-*N*-biosidase (GH20 and GH136), and GNB/LNB phosphorylase (GH112) have been described to participate in the utilization of HMOs and other milk-based substrates (i.e., lactose and GOS) [28,36,37,38,39,40,41,42,43,44,45]. Only *B. bifidum* and strains of *B. longum* that encode an extracellular Lacto-*N*-biosidase (GH136) employ the external strategy [21,43,46,47,48].

Although the gene repertoires and applied strategies are strongly species-specific, the growth rates, efficiency rates, preferences [27], and ranges of the utilized HMOs can differ among strains of the same species [15,19,20,26,27,31,33,49]. For instance, the fucosylated HMO (FHMO) consumption rates vary significantly between species, and generally all *B. infantis, B. bifidum*, and *B. kashiwanohense* strains utilize FHMOs. However, recent genomic analyses have revealed that the gene sets for FHMO degradation are also distributed in less than 10% of *B. longum*, *B. breve*, and *B. pseudocatenulatum* strains [17,21,50], providing strains with the capability to utilize FHMOs, likely through inter-species horizontal gene transfer [17].

Nevertheless, our understanding of the mechanisms that different strains from the same species apply to assimilate HMOs remains incomplete. The objective of this study was to assess the effects of strain-level genomic variation in HMO utilization genes (GH, transporters, and regulatory and carbohydrate handling) on the HMO utilization potential across human-associated bifidobacteria. For this, we tested the growth of 130 strains from 10 species (*B. adolescentis* (13), *B. angulatum* (2), *B. animalis* (4), *B. bifidum* (14), *B. breve* (8), *B. catenulatum* (8), *B. longum* subsp. *infantis* (8), *B. longum* subsp. *longum* (67), *B. pseudocatenulatum* (5), *B. scardovii* (1)) on 6 HMOs (LNT, LNnT, 2′FL, 3FL, 6′SL, 3′SL), HMO building block monosaccharides, and backbone di-saccharide lactose. Understanding the strain-specific HMO consumption pathways will provide a better mechanistic rationale for the development and optimization of new early-life microbiome-modulating products.

## 2. Materials and Methods

### 2.1. Bacterial Strains and Growth Conditions

The 130 *Bifidobacterium* strains used in this study (Table S2) were previously purchased from the American Type Culture Collection (ATCC), isolated from a commercial probiotic product, or derived from the Chr Hansen Culture Collection (Chr Hansen, Denmark). The latter strains were isolated from feces (infants and adults) and are depicted with a Bif number. The bifidobacteria were routinely grown on Man–Rogosa–Sharpe (MRS; BD Difco, New York, NY, USA) medium, supplemented with 0.05% *w*/*v* L-cysteine-HCl (Merck KGaA, Darmstadt, Germany) (MRS-Cys) and incubated at 37 °C. All incubations were performed in airtight AnaeroPack boxes (Mitsubishi Gas Chemical Company, Inc., Tokyo, Japan) with AnaeroGen (Thermo Scientific, Oxoid, MA, USA) sachets added to create an anaerobic atmosphere.

### 2.2. Growth on Carbohydrate Sources

The six HMOs tested (2’-*O*-fucosyllactose (2′FL), 3-*O*-fucosyllactose (3FL), lacto-*N*-tetraose (LNT), lacto-*N*-neotetraose (LNnT), 3’-*O*-sialyllactose sodium salt (3′SL), and 6′-*O*-sialyllactose sodium salt (6′SL) were kindly donated by Glycom A/S Denmark, through their scientific donation program. In addition, the 5 HMO building block monosaccharides (glucose, galactose, *N*-acetylneuraminic acid, l-fucose, *N*-acetyl-d-glucosamine) and the backbone di-saccharide lactose were all purchased from Merck. For the evaluation of growth on the 12 carbohydrates as the sole carbon source, the strains were cultured in modified MRS (mMRS) medium, which was manually prepared to obtain the following composition: tryptone (10.0 g/L), yeast extract (5 g/L), sodium acetate trihydrate (5 g/L), K_2_HPO_4_ (2.6 g/L), di-ammonium hydrogen citrate (2.0 g/L), Tween-80 (1 g/L), l-cysteine-HCL (0.5 g/L), MgSO_4_·7H_2_O (0.2 g/L), MnSO_4_·H_2_O (0.05 g/L). Prior to autoclaving at 121 °C for 15 min, the medium was adjusted to pH 6.9. All carbohydrates were added directly to the mMRS medium (mMRS-HMO) at a final concentration of 2% (*w/v*), which was subsequently filter-sterilized (0.2 μm). All strains were cultured in biological triplicates. For the growth assays, the strains were first grown overnight at 37 °C in MRS-Cys. Ten-fold dilution series were prepared from the overnight cultures and incubated overnight under the same conditions with fresh medium. For each strain, late exponential–early stationary-phase cells were selected based on their optical density at 600 nm (OD_600nm_). These were washed twice in large volumes of mMRS medium and normalized to OD_600nm_ = 1.0 in mMRS, prior to inoculation. The growth experiments were performed in triplicate in 96-well microtiter plates with 180 µL of mMRS-HMO and 20 µL of bacterial cell suspension. Furthermore, each experiment contained a well with MRS-Cys as the positive control, mMRS-HMO without bacteria as the negative control, and mMRS medium as the background growth control. The microtiter plates were incubated at 37 °C under anaerobic conditions. The bacterial growth was measured at OD_600nm_ with a Synergy H1M (Biotek, Winooski, VT, USA) plate reader after 96 h of growth.

### 2.3. Genome Sequencing, Assembly, Annotation, and GH Identification

The bacteria were grown overnight in MRS-Cys and the pellets were dissolved in DNA/RNA Shield (Zymo research Europe, Freiburg, Germany) and subsequently shipped to BaseClear BV, Leiden, The Netherlands, where the paired-end sequencing was performed on Illumina platforms.

The draft genomes were de-novo-assembled with SPAdes v3.12.0 [51] using the ‘--careful’ setting, and predictions of putative open reading frames (ORFs) and annotations based on BLASTP were performed with PROKKA v1.14.5 [52]. As Illumina-based genome sequencing does not allow the complete closure of bacterial genomes, we assessed the genome completeness using CheckM2 v1.0.2 [53]. The glycosyl hydrolases were predicted and annotated based on similarity to the Carbohydrate-Active enZYmes (CAZy) (http://www.cazy.org/) database [54]. The counts of glucosyl hydrolase (GH) families were extracted using custom Python scripts and subsequently reduced to only the GH families associated with HMO degradation.

### 2.4. Homology Searches for HMO Utilization Genes 

Queries of nucleotide sequences based on the literature were retrieved from full genome sequences of bifidobacteria deposited in the NCBI reference sequence database, accessed on 1 July 2023 (https://www.ncbi.nlm.nih.gov/), and the JGI repository accessed on 18 May 2022 (https://genome.jgi.doe.gov/portal/). The accession numbers are listed in Table S1. The query nucleotide sequences of the *B. scardovii* GHs were taken from the corresponding genome. The prevalence and similarity rates of the HMO utilization genes in the 130 draft *Bifidobacterium* genomes were examined via a BLAST analysis (BLAST + v2.9.0) using the Resfinder engine v3.2 [55], with the following criteria: identity >50%, query coverage >50%. We ran the analysis with different thresholds to estimate the importance, and going lower or higher did not affect the main results. The heatmaps were generated with R version 4.2.0 [56] using the ComplexHeatmap v2.11.2 package [57].

## 3. Results and Discussion

### 3.1. Genome Sequencing

To identify the HMO-degrading machinery of the bifidobacteria, we performed genome sequencing and assembly and carried out a uniform open reading frame (ORF) prediction of 130 isolates. Illumina-based genome sequencing does not allow the complete closure of bacterial genomes; therefore, we assessed their genome quality using genome completeness (99.9 ± 0.2%), contamination (0.36 ± 0.68%, contig N50 (267k ± 270k nt), and L50 (5.3 ± 2.8) values, which all indicated that the genomes were of high quality and suitable for the subsequent analyses. The numbers of predicted ORFs ranged from 1550 for a *B. animalis* to 2607 for a *B. infantis*. The average G + C content was 59.8 ± 1.6%, and the values ranged from 55.9% for a *B. catenulatum* to 64.8% for *B. scardovii*. The average genome size was 2.32 ± 0.19 Mbp, whereby *B. scardovii* had the largest genome (3.13 Mbp) and *B. animalis* the smallest (1.92 Mbp) (Table S2). As an additional quality check based on biological genomic features, we calculated the previous genomic characteristics per species (Tables S3–S5). All metrics were in accordance with the published reference statistics for each species [19,26,58,59,60,61].

### 3.2. Common HMO Degradation Machinery of Human Bifidobacteria

#### 3.2.1. GHs Involved in Degradation of Neutral Type I and II Chain HMOs

A heatmap summarizing all genes found in all genomes is supplied as Supplementary Figure S1. When reporting the statistics and prevalence of the common HMO-degrading genes in this chapter, we excluded *B. animalis* and *B. angulatum*, as they are not prototypical human species or do not degrade HMOs. However, their data are supplied as Supplementary Information.

Three GH families are involved in the degradation of the glycosidic bonds in type I and II HMO backbone structures—β-1,4 galactosidase (GH2), β-1,3-galactosidase (GH42), and β-*N*-acetylglucosaminidase (GH20). We found homologs of the cognate LNT β-1,3-galactosidases (GH42) from *B. breve*, Bbr_0529 (lntA) [32], and *B. infantis* Blon_2016 (Bga42A) [37] in 100% of the strains from each species, except for *B. adolescentis* (prevalence rate of 38%) (Figure S1). These enzymes are highly active on LNT but have shown cross-reactivity towards Lac, LNB, and LNnT for Blon_2016 and LNT, LNnT, and lactose for Bbr_0529. The functionally equivalent orthologs in *B. bifidum* and *B. longum* display the same dual activity towards both type I and II chains [62]. Their prevalence across all tested infant-derived bifidobacterial species supports their relevance in HMO degradation.

In contrast, the prevalence of β-1,4-galactosidases (GH2) was more species-specific. These enzymes are only active towards β-1,4-linked Gal in lactose and type 2 chains. The best conserved GH2 β-1,4 galactosidase, Blon_2334 [37] from *B. infantis*, was absent in *B. breve* and *B. bifidum* and highly prevalent in *B. catenulatum* and *B. pseudocatenulatum* (100%), *B. infantis* (93%), and *B. longum* (76%). The second most prevalent gene was bbr1552 (lacZ6) (lntA) from *B. breve* [32], which showed 100% prevalence rates in both *B. breve* and *B. bifidum* but was absent in all other strains (Figure S1).

The most prevalent β-*N*-acetylglucosaminidase (GH20), Blon_0732 from *B. infantis* [45], was 100% prevalent in *B. infantis* and *B. longum*. The version found in 100% of the *B. breve strains*, Bbr_1556 (NahA), was homologous to a different *B. infantis* GH20 enzyme, namely Blon_0459. In contrast, *B. pseudocatenulatum* (100%) and *B. catenulatum* (56%) shared a unique GH20 gene, BBPC_1688, not found in any other species. The prevalence of β-*N*-acetylglucosaminidases across all tested infant-derived bifidobacterial species supports their relevance in HMO degradation. However, their overall prevalence rate was lower than for LNT β-1,3-galactosidases, as in both *B. pseudocatenulatum* and *B. catenulatum* they were located inside LNB/LNT utilization clusters, which were sometimes entirely absent from the latter species (Figure S1).

#### 3.2.2. Lnp Locus

The Lnp gene cluster is responsible for the import of mainly LNB by the ABC transporter (gltABC) and subsequent phosphorolysis by GH112 GNB/LNB phosphorylase (lnpA), releasing Gal-1-P,GlcNAc/GlcNAc-6-P through the modified Leloir pathway [31,38,49,63,64]. The complete *lnp* locus was prevalent in 100% of the prototypical infant species *B. longum*, *B. infantis*, *B. bifidum*, and *B. breve*. Interestingly, in *B. longum*, we found two different versions of this gene cluster, on which we further elaborate in the species-specific chapter.

In contrast, all *B. pseudocatenulatum* and 4 out of 8 *B. catenulatum* strains encoded the gltABC transporter, although this was combined with LNB/LNT utilization machinery (nagA, nagB, and a GH20 GH) instead of the conventional *lnp* cluster layout that combines this transporter with the modified Leloir-like pathway, like the other infant bifidobacteria [65]. *B. adolescentis* did not encode an lnp cluster or gltA (Figure S1).

#### 3.2.3. Fucosyllactose–Fucose Utilization Loci

The *Bifidobacterium* fucose and FHMO utilization loci identified outside the typical FHMO utilizers *B. infantis* and *B. bifidum* are similarly organized. They consist of intracellular fucosidases (AfcA or AfcB), fucose catabolism genes, and an ABC transporter. The SBPs of the ABC transporter responsible for the intake of fucosylated (FL) HMOs are classified into 2 types, FL1-BP and FL2-BP. These are further classified into 4 clades based on their substrate specificity, comprising cluster 1-IV (FL1-BP) and clusters 2-I, 2-II, and 2-III (FL2-BP). These transporters are exclusively distributed in bifidobacterial genomes. Transporters belonging to cluster 1-IV can import 2′FL and 3FL, whereas clusters 2-I and 2-II can import 2′FL, 3FL, lactodifucotetraose (LDFT), and lacto-*N*-fucopentaose I (LNFP I). Cluster 2-III can take up the widest range of FHMOs—2′FL, 3FL, LDFT, LNFP I, LNFP II, and lacto-*N*-difucohexaose I/II (LNDFH I/II). The substrate specificity of the FL transporter type in the FHMO cluster corresponds to the presence of corresponding fucosidase genes, i.e., transporters with a narrow substrate specificity are paired with a GH95 AfcA homolog 1,2-α-l-fucosidase, while transporters with wider substrate specificity rates are paired with a GH95 and complementary GH29 AfcB homolog 1,3/4-α-l-fucosidase to cover the larger range of imported FHMOs [19]. The additional GH29 comes paired with a l-fucose mutarotase FucU. Genomic analyses have revealed that these gene sets for fucosylated HMO degradation are distributed in almost all strains of *B. kashiwanohense*, although in less than 10% of strains of *B. longum*, *B. breve*, and *B. pseudocatenulatum* [17,21,50]. In this dataset, we identified versions of this complete FHMO cluster in one *B. pseudocatenulatum* strain but none in 67 B. longum strains. Interestingly, in all 8 *B. breve* strains we only found partial, non-functional gene sets.

### 3.3. Species-Specific HMO Degradation Patterns and Genetic Machinery

Despite these shared genetic characteristics, most gene sets were species-specific in their homology, content, and organization, exemplified by the *B. infantis* 43,000 kb H1 HMO cluster, the extracellular *B. bifidum* GHs, and the *B. breve lnt* and *nah* loci. However, we also observed within-species strain-level genetic variation, of which the extent differed between species. The HMO degradation profiles were mostly strain-specific due to the similarity in the utilized HMOs, except for *B. infantis* and *B. bifidum*, strains whose degradation patterns were similar within species and highly divergent from other species. Due to the clear species-specific genotype and strain-specific phenotype, we performed species-specific gene trait matching to determine the impact of genetic variation on HMO utilization.

#### 3.3.1. *B. breve*

##### Results

All *B. breve* strains grew well on LNT, while 3 out of the 8 strains did not grow on LNnT. All 8 strains contained at least one member of the required GH families for LN(n)T/G degradation (GH2 (lacZ2/Z6), GH42 (nahA), and GH20), as well as one GH112 LNB-GNB phosphorylase, GH33 α-sialidase, and GH95 1,2-α-fucosidase. As the HMO GH profile was conserved in all strains, the reasons for these phenotypical differences must be in the auxiliary HMO degradation machinery.

Both the *lnt* and *lnp−glt* loci were extremely well conserved in all strains. However, we observed small variations in the LN(n)T SBP (nahS) and transcriptional response regulator nahR from the *nah* locus. One group encoded a nahS gene with 97% identity versus 99% identity, as compared to the gene from strain UCC2003. This different SBP correlated with the lower overall growth on LNnT. Additionally, we observed some small changes in the nahR transcriptional regulator, although this did not seem to have a large effect on the final OD_600_ value; however, it might affect the response time and growth rate in vivo.

**Figure 1:**
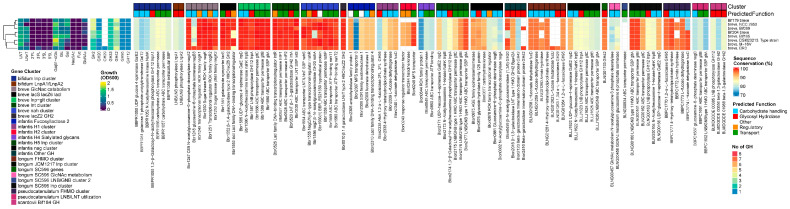
*B. breve*, from left to right: heatmap of growth on HMOs and building blocks (OD_600_); number of predicted GH genes and prevalence and sequence conservation rates of HMO utilization genes. The query gene clusters and predicted global function are color-coded on top, while the locus tag and protein name are shown at the bottom.

Despite the presence of fucosidases, no strains grew on 2′FL/3FL. All tested strains encoded the complete fucose catabolism machinery and fucosidases yet interestingly lacked the complete FL ABC transporter. To better evaluate the prevalence of this genotype, we additionally analyzed 7 *B. breve* genomes from our database that were not part of the HMO screening presented here. These strains displayed the same genetic configuration. Lastly, all strains encoded a GH33 α-sialidase but did not grow on 3/6′SL (Figure 1).

##### Discussion

In *B. breve* UCC2003, the *lnt* (Bbr_0526-0530) and *nah* (Bbr_1554-1560) loci are responsible for the uptake and breakdown of LN(n)T. The *lnt* locus transporter is specific for the uptake of LNT and intracellular hydrolysis of both LNT and LNnT, releasing Gal and producing lacto-*N*-triose. The *nah* locus transporter takes up both LNT and LNnT and contains a GH20 β-*N*-acetylglucosaminidase that cleaves GlcNAc from lacto-*N*-triose, leaving lactose, which is degraded by redundant enzymes; GH42 LNT β-1,3-galactosidase; nahA (Bbr_1556) with dual activity towards β-1,4-linkages in lactose; and the GH2 β-1,4-galactosidases lacZ2 and lacZ6 (Bbr_0010 and Bbr_1552, respectively), located elsewhere in the genome [32]. The *nag* locus (Bbr_1247-1252) is involved in the multistep metabolism of GlcNAc. The *lnp-glt* locus (Bbr_1585-1590) is responsible for the internalization and subsequent phosphorolysis of free LNB, releasing Gal-1-P,GlcNAc/GlcNAc-6-P [32].

The lack of growth on LN(n)T by 3 of the included 8 strains was discordant with a group of 24 strains that all showed good growth on both substrates [20]. However, inconsistencies in the growth phenotypes of strains have been observed previously, such as growth [20] and lack of growth [17] on LNnT by the type strain DSM20213^T^. Nevertheless, all tested strains contained at least one member of the required GH families for LN(n)T degradation and one GH112 LNB-GNB phosphorylase, GH33 α-sialidase, and GH95 1,2-α-fucosidase, in concordance with these being reported as part of the *B. breve* core glycobiome [58]. *B. breve* is known to possess broad but variable saccharolytic capacity, although the GHs specific for HMO degradation seem to be well conserved.

One strain encoded an extra GH29 α-1-3/4-fucosidase. This gene was also identified in 4 out of 24 strains, of which 15 were isolated from 3–4 month-old exclusively breastfed term infants from the USA [20], and 0 out of 13 isolates from Japanese infants [21], making this a fairly rare genotype. Despite the presence of fucosidase, no strains grew on 2′FL/3FL. Nevertheless, this phenotype has been reported for *B. breve* strains encoding the FHMO locus with an FL2-II/AfcA and FL1-IV/AfcA configuration [21]. All tested strains (and 7 of the additionally analyzed genomes) encoded the complete fucose catabolism machinery and fucosidases yet interestingly lacked the complete FL ABC transporter. The presence of a strain with the additional GH29 AfcB combined with a FucU gene hints at *B. breve* strains with wider HMO assimilation phenotypes that might still be discovered. Interestingly, the presence of this partial cluster in our dataset is in contrast to other strains where either the whole cluster was present or absent [17,19,50].

As the *B. breve* strains with FL-SBs were all previously isolated from Japanese infants from 2 studies [21,66], we wondered whether a 2′FL-consuming strain isolated from a North American infant (SC95) would also display this genomic feature. However, we found the same partial transporter-deficient genotype. This discrepancy still remains unexplained, although the lack of growth on 3FL for this strain, which should be degraded by the additional GH29 α-1-3/4-fucosidase in its genome, might hint at the use of a novel transporter that only imports 2′FL.

Although all strains encoded a GH33 α-sialidase, none grew on 3/6′SL, which is a common feature, as *B. breve* strains have been shown to consume acidic HMOs, although they prefer more complex structures such as LSTb and monosialyllacto-N-hexaose (S-LNH) [20]. Similarly, one strain that did not grow on 2′FL did utilize larger fucosylated HMOs from a complex human milk mixture [20]. This also points to the previously mentioned presence of novel undiscovered transporters or yet unknown SBP specificity for *B. breve* strains. In conclusion, the pan genome based on 73 *B. breve* strains has been shown to be open and approaching saturation. The observed genetic diversity included genes involving various dietary- or host-derived carbohydrate utilization capabilities [58]. This allows *B. breve* to utilize dietary substrates associated with infancy up to weaning (HMOs, lactose, and plant-derived carbohydrate sources). Additionally, the tightly controlled system for the transcriptional regulation of HMO metabolism allows *B. breve* strains to rapidly switch their metabolic processing to and from HMOs, lactose, and plant-derived carbohydrate sources, which is a regular occurrence during weaning. This variability in dietary carbohydrates usage seems to extend to the HMO consumption profiles in the case of *B. breve*. The cores of the transporters and GHs are sufficient for the degradation of LN(n)T; therefore, the majority of strains grow well on both. However, the apparent variation in the degradation of more complex structures points to the prevalence of yet unknown acidic and fucosylated HMO transporters in combination with a GH29 α-1-3/4 fucosidase.

#### 3.3.2. *B. longum* subsp. *infantis*

##### Results

All *B. infantis* strains grew well on all substrates, although the OD_600_ values were generally lower on 3′SL and 6′SL. In our set of 8 strains, two out of three GH20 β-*N*-acetylglucosaminidases were conserved in all strains, namely Blon_2355 located in H1 and Blon_0732 located outside of H1. Blon_0459 was missing from 2 strains. Both the GH42 LNT β-1,3-galactosidase Bga42A/Blon_2016 and GH2 Bga2A/Blon_2334, located in H1, were conserved in all strains. In contrast, the prevalence of other GH2/GH42 β-galactosidases was strain-specific, although these are active on β-1,4-gal in GOS rather than LN(n)T [62,67]. Four out of the five previously reported fucosidases in the type strain ATCC15697^T^ were conserved in all strains, whereas the final fucosidase belonging to GH151 (Blon_0346) was missing in three out of six strains. All strains encoded GH33 α-sialidases, NanH2/Blon_2348 located in H1, and NahH1/Blon_0646 located in H4. All strains also contained one GH112 GNB/LNB phosphorylase located in the *lnp* (H5) cluster.

**Figure 2:**
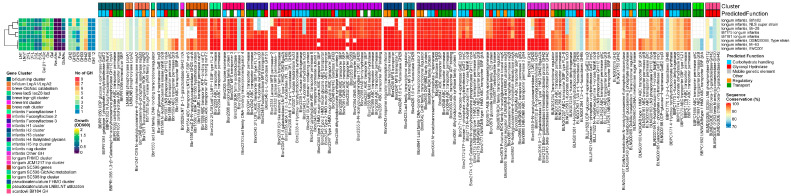
*B. infantis*, from left to right: heatmap of growth on HMOs and building blocks (OD_600_); number of predicted GH genes and prevalence and sequence conservation rates of HMO utilization genes. The query gene clusters and predicted global function are color-coded on top, while the locus tag and protein name are shown at the bottom.

All strains contained at least one GH for the internal sequential degradation of both type I and type II cores, FHMOs, and acidic HMOs; therefore, the specialization towards specific HMOs in our set is not based on GH profile alone but rather the complement of SPBs can predict the spectrum of available substrates and is responsible for phenotypical differences in vitro (and likely in vivo) (Figure 2).

Considering the complete HMO degradation machinery, we could broadly divide our strains into four genetic configurations based on the presence or absence of (partial) genetic loci. Group I had retained the most complete utilization machinery (M-63, EVC001, ATCC15697^T^). The three other groups missed the complete *lnp*/H5 transporter (gltABC). Group II additionally missed nahS (Bif175, Bif181) and group III missed both nahS and fucosyllactose cluster 3 (Bi-26). Lastly, group IV missed fucosyllactose cluster 3 and partially H2 (NLS superstrain, Bifin02).

Taking group I as a reference, as these strains retained the most complete utilization machinery, the following SBPs were missing in other groups: group II, Blon_2177/gltA, nahS; group III, Blon_2177/gltA, nahS, FL1-IV BP; group IV, Blon_2177/gltA, FL1-IV BP. Additionally, some strains also missed at least one SBP from H1, with affinities for either type II chains or GNB (Blon_2344, Blon_2351, Blon_2352). Nevertheless, five out of six of the aforementioned SBPs had one or more related SPBs, with similar substrate specificity remaining in each strain. Blon_2344 and Blon_2347 specifically recognize type 2 glycans and nahS recognizes LN(n)T [50]. Where Blon_2344 or nahS was absent, Blon_2347 remained. Blon_2351, Blon_2350, and Blon_2354 specifically detect GNB [50]. When Blon_2351 was absent, Blon_2350 and Blon_2354 remained. Likewise, the absence of 2′FL/3FL-specific FL1-II BP (Blon_0343) was always compensated for by FL2-IV (Blon_2202), which has overlapping but wider substrate specificity (2′FL/3FL/DFL/LNFP−I) [68]. The only exception was gltA in *lnp*/H5 (Blon_2177), which recognizes LNT/GNB/LNB and a wide range of larger type 1 HMOs [27,69].

##### Discussion

The type strain *B. longum* subsp. *infantis* (*B. infantis*) ATCC15697 ^T^ is the archetypical HMO-utilizing bacterium, which possesses several unique gene clusters, of which a 43 kb contig, the HMO cluster I (H1), encodes the most elaborate HMO uptake and intracellular degradation machinery among the bifidobacteria [70]. It uses a multitude of transporters to directly internalize a large range of neutral, acidic, and fucosylated HMOs and degrade them intracellularly. In ATCC15697^T^, the HMO-degrading machinery is spread over several loci; H1 includes genes encoding for a fucose catabolic operon (Blon_2337-2340) [17]; several SBPs specific for the uptake of type II chain HMOs or GNB; and one copy each of GH2, GH20, GH29, GH95, and GH33 genes. H2 and H3 encode genes involved in fucose uptake and catabolism, respectively. H4, a sialidated glycan catabolism cluster, was originally thought to be involved in HMO utilization [71], although is now considered to be used for sialylated glycans other than HMOs (e.g., milk glycopeptides or sialylated mucins) [72]. H5, the *lnp* cluster (Blon_2171-2177), encodes the modified Leloir-like pathway for processing LNB/GNB and an ABC transporter + SBP with broad affinity for GNB and LNB, type 1 HMOs (LNT, LNH, LNO), and related glycans, including sialylated LNT and LNnT [27,50,65,73]. Three more fucosyllactose clusters encode for a(n): an ABC transporter with FL2-II SBP (blon2202-2203); an ABC transporter with FL1-IV SBP combined with an GH151 α-L−fucosidase (GH151) (Blon_341-346); the fucose catabolic pathway (Blon_2306-2310). Lastly, the *nag* cluster encodes the GlcNAc catabolic pathway and an ABC transporter + SBP with high affinity for GNB (Blon_879-885). The essential GH42 Bga42a LNT β-1,3-galactosidase is located outside these clusters (Blon_2016).

The growth profiles of our strains confirmed the previous work, showing that the degradation capabilities of individual HMOs were comparable (i.e., all strains grow well on all tested HMOs), although small differences have been reported, such as one out of 21 strains displaying poor growth on 6′SL [30]. Nevertheless, at a deeper level, strains have been shown to differ in both their substrate efficiency (max OD_600_) and growth rates on individual HMOs [27,33,66].

The aforementioned different configurations of HMO degradation gene sets suggest specialization towards specific or more broad HMO categories. Some strains have lost the unique gltA from *lnp*/H5 (Blon_2177) for the import of larger type 1 HMOs [27,69], whose absence likely renders these strains incapable of internalizing these HMOs whole. In contrast, none of our tested strains (or other strains) have been shown to miss both FL1-II BP and FL1-IV BP, suggesting that the competitive advantage of FHMO utilization is indispensable for *B. infantis* and is not exchanged for the broader range of complex HMOs that gltA offers. Therefore, strains within the subspecies *B. infantis* may be broadly divided into generalists or those focused more on the utilization of smaller (fucosylated) HMOs.

The observed configuration (SBPs missing from H1 and the absence of partial or complete genetic loci) is concordant with research from 2010, which used *B. infantis* strains isolated before 1990, before this subspecies was commercially produced as a probiotic. Furthermore, the absence of the gltABC transporter was observed in <25% of 21 publicly available *B. infantis* genomes [49], as well as in a recently isolated native strain from Bangladesh [74]. This strongly suggests that this feature is prevalent in natural *B. infantis* populations, rather than the result of gene reduction in the production of probiotics, as previously suggested [26].

Interestingly, these 4 genotypical configurations coincide with 4 groups that were based on the total *B. infantis* genome size. The group that included the type strain had an average genome size of 2796 kb, while another group including Bi-26 displayed a 7.3% smaller average genome size (2606 kb). Part of that genome size difference was attributed to selected gene loss in Bi-26, largely of various transporters and glycosidases, which likely reflects adaptation to the utilization of specific carbohydrates [27]. However, Bifin02, with a similar configuration of its HMO clusters as Bi-26, has a genome size of 2758 kb (closed circular genome), indicating that other genes are also responsible for these genome size differences.

Recently, regulatory mechanisms for HMO degradation in the generalist (gltA+) *B. infantis* ATCC15697^T^ were identified. GlcNAc and its phosphorylated derivatives, GlcNAc-6P and GlcNAc-1P, were identified as potential nagR transcriptional effectors, suggesting that the release of GlcNAc during the internal degradation of any GlcNAc-containing glycans by GHs (e.g., LN(n)T, and in particular fucosylated HMOs (e.g., LNFP I) and milk N-glycans), results in the upregulation of nagR-controlled genes. These de-repress the nag and H1 cluster, including genes encoding LNT and LNnT transporters, as well as the *lnp*/H5 cluster, explaining the similarity of the transcriptomic responses of *B. infantis* to LNT and LNnT in vitro [30,33]. In contrast, in response to fucosyllactoses (2′FL, 3FL, and DFL), all top upregulated genes occurred in alternative operons outside H1 (fucosyllactose cluster 1–3) [30,70], indicating that dedicated pathways for fucosyllactose utilization have evolved in *B. infantis*.

Therefore, some strains seem to have evolved to use certain pathways more efficiently, both via the gene deletion of gltABC, as observed in our dataset, and by differing in their global regulatory responses to HMO structures (suggesting changes in global regulatory response networks) [27,69]. This has previously been shown in the group III strain Bi-26 (missing gltABC, nahS, and fucosyllactose cluster 3). Compared to the generalist type strain, small fucosylated HMOs exerted far-reaching regulatory roles comparable in effect size to that of lactose—the preferred carbon source of *B. longum* [75] and potentially one of its global regulators [76,77]. In contrast, the type strain perturbed the expression of a much smaller complement of genes. This led to higher growth rates of Bi-26, potentially providing a competitive advantage over other *B. infantis* strains in infants breastfed with milk high in 2′FL or 3FL. Another way to gain a competitive advantage would be to employ different sequential preference utilization profiles.

Taken together, these findings demonstrate major strain-specific adaptations to the efficient utilization of FLs via two routes: a different configuration of the HMO degradation machinery caused by the gene deletion of the transporters GltABC and nahS to restrict the uptake range of (neutral) HMOs and deletion of the redundant FHMO transporters (FL1-IV) with lower substrate specificity; a change in global regulatory response networks to different HMOs. Other configurations might lead to varying efficiencies (more biomass from the same substrate), response times, growth rates, and sequential preferences for other single HMOs. Conversely, the group I strains, including the type strain, have maintained all genes needed for the metabolism of all HMOs, as well as genes more likely related to mucin usage. These differences also result in altered metabolic outputs or shifts in the ratios of metabolic end-products, including metabolites used in cross-feeding with other microbes, such as 1,2-propanediol and fucose [78], which eventually would impact ecosystem dynamics.

While most of the functionality is retained through genetic redundancy, the genetic specialization of some *B. infantis* strains (and the complex NagR regulon structure in the generalist *B. infantis* ATCC15697^T^) likely reflects the evolutionary adaptation to the simultaneous utilization of multiple HMOs. This suggests that the use of rationally formulated HMO mixtures rather than individual oligosaccharides as prebiotics may be a more efficient solution for the selective stimulation of *B. infantis* growth in the neonatal gut, as it considers the nuanced regulatory mechanisms and physiology of the target organism [65].

#### 3.3.3. *B. longum* subsp. *longum*

##### Results

Almost all of the tested 67 *B. longum* strains grew moderately to well on LNT only, whereas a few strains showed minor growth on LNnT. We found copies of GH42 LNT-β-1,3-galactosidase (BLNG_01753 [31], a homolog of Bga42a/lntA) and GH20 β-N-acetylglucosaminidase [79] (a homolog of Blon_0732) in all strains. However, BLNG_00015 [31], a homolog of GH2 β-1,4-galactosidase Bga2A, was only present in 51 out of 67 strains (76%). Therefore, all strains encoded the full complement of genes to internally degrade LN(n)T, as Bga42a/lntA is also effective on β-1,4-linked-gal. Despite all strains encoding one GH112 GNB/LNB phosphorylase [38], we identified two different versions of the gene—one related to BLLJ_1623 [73] from the adult isolate and type strain JCM1217^T^, while the other was more similar to BLNG_00163 [31], from the infant isolate SC596. Lastly, the *B. longum*-specific extracellular membrane-bound GH136 lacto-N-biosidase (lnbX) was present in 43 out of 67 (64%) strains, and the co-occurrence rate with the chaperone needed for the proper folding and function of lnbX (lnbY) [80] was 100%.

**Figure 3:**
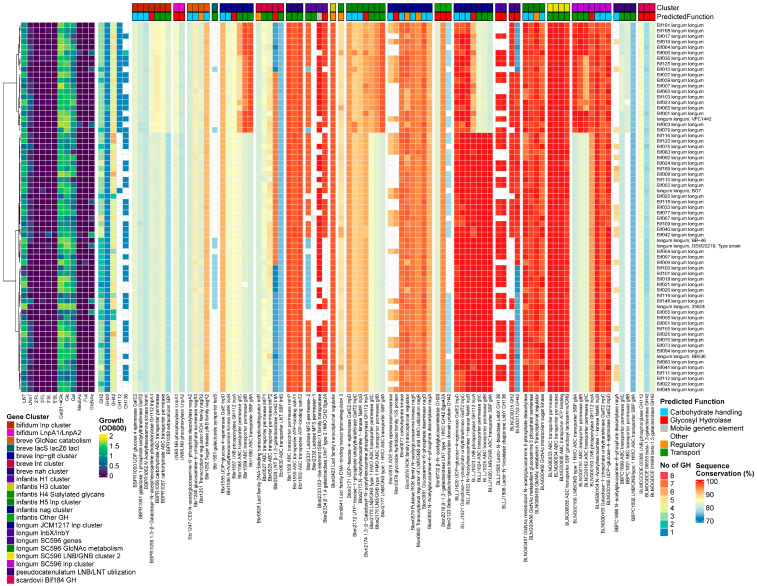
*B. longum*, from left to right: heatmap of growth on HMOs and building blocks (OD_600_); number of predicted GH genes and prevalence and sequence conservation rates of HMO utilization genes. The query gene clusters and predicted global function are color-coded on top, while the locus tag and protein name are shown at the bottom.

The prevalence rate of the complete *lnp* locus was 100%. However, the gltABC transporter was also present in two versions; here, 47 out of 67 (70%) strains encoded a homolog to the adult isolate JCM1217^T^ (BLLJ_1624-1626), while the rest encoded a homolog to the infant strain SC596 (BLNG_00160-00162). All genomes also contained a well-conserved group of genes homologous to the *B. infantis nag* cluster, although the additional gltFGH (GNB) transporter was missing in all *B. longum* strains (Figure 3).

##### Discussion

Despite differences in the prevalence rates of most bifidobacterial species with ageing, *Bifidobacterium longum* subsp. *longum* (*B. longum*) is widely distributed in the guts of infants, adults, and elderly subjects [12,81]. Strains consuming LNT are thought to contain the SBP of the ABC transporter (GNB/LNB-BP (GltA)), as well as LNT-degrading intracellular enzymes, including GH42 LNT β-1,3-galactosidase, GH20 β-N-acetylglucosaminidase, GH2 β-1,4-galactosidase, and GH 112 GNB/LNB phosphorylase [31,49]. Specific *B. longum* strains additionally employ an extracellular glycosidase-dependent strategy, and these strains contain a membrane-bound extracellular GH136 lacto-N-biosidase (LnbX), which needs a chaperone for proper folding and function (lnbY) [80]. LnbX can degrade LNT to LNB and lactose [80], whereafter LNB is imported by GL-BP (gltA) and subsequently phosphorolysed by GH112 LnpA [38], releasing Gal-1-P,GlcNAc/GlcNAc-6-P through the modified Leloir pathway encoded by the *lnp* cluster [31,49].

Most of our tested strains grew moderately to well on LNT, while only a few displayed minor growth on LNnT, in line with previous observations [31]. None of the tested strains grew on 2′FL/3FL, and as expected we found no homologs to any of the genes for FHMO uptake, hydrolysis, or L-fucose catabolism encoded by the FHMO locus [17,19,21]. The prevalence rate of this cluster in *B. longum* was only 3% in 151 genomes in public repositories [49], so its absence in our set of 67 strains was to be expected.

We confirmed a 100% prevalence rate for one GH42 LNT-β-1,3-galactosidase (BLNG_01753) [31] (a homolog of Bga42a/lntA) and one GH20 β-*N*-acetylglucosaminidase [79] (a homolog of Blon0732), as previously reported [49]. However, BLNG_00015 [31] (a homolog of GH2 β-1,4-galactosidase Bga2A) was only observed in 51 out of 67 (76%) strains, which was significantly lower than the 100% result previously reported [49]. Therefore, all strains encoded the full complement of genes to internally degrade LN(n)T, as Bga42a/lntA prefers terminal β-1,3-linked-gal; however, it is also effective on β-1,4-linked-gal, although having the specialized Bga2A might be more efficient. The 43 out of 67 (64%) prevalence rate of the extracellular membrane-bound GH136 lacto-*N*-biosidase (lnbX) was significantly higher than the 38% previously reported in 151 *B. longum* strains [49].

Although the prevalence rate of the complete *lnp* locus was 100%, interestingly the gltABC transporter and GH112 GNB/LNB phosphorylase were present in two versions. Here, 47 out of 67 (70%) strains encoded homologs to the adult isolate JCM1217^T^ (BLLJ_1623-1626), while the rest encoded homologs to the infant strain SC596 (BLNG_00160-00163). Although gltA from JCM1217^T^ can bind LNT, its binding affinity is approximately 1000x and 100x lower than for GNB and LNB, respectively [73], and it is not considered to import LNT, as the disruption of lnbX in another adult strain with the same gltA (100% nt identity), JCM31944, eliminated the growth on LNT [82]. In contrast, gltA from the infant strain SC596 (BLNG_00160) displayed similar affinities for type 1 HMOs as its homolog in *B. infantis* (high affinity for LNB, GNB, LNT, and other structurally related glycans, such as sialyl LNT, and even several complex bi- and tri-antennary *N*-glycans, such as those found in milk and intestinal glycoproteins [31]).

All genomes also contained genes homologous to the *B. infantis nag* cluster, encoding the GlcNAc catabolic pathway (BLNG00457-4060), without the additional gltFGH (GNB) transporter. When present, both the *lnp* and *nag* (nagK, nagB, and nagA) clusters are organized in the nagR regulon, as previously mentioned for other *Bifidobacterium* species. In the single *B. longum* strain lnbX+ that was tested, this gene was additionally under the control of nagR [65].

Therefore, the main differences in the LNT-utilizing machinery were in the presence and absence of lnbX and the 2 versions of the *lnp* cluster. The prevalence of lnbX was 64% in all strains and 55% and 68% in combination with the infant (BLNG_00160) and adult (BLLJ1626) gltA types, respectively. In all cases, the growth of the lnbX+ strains was better than for lnbX- (OD_600_ values in all strains: 1.16 vs. 0.89, *p* = 0.0003; combined with BLNG_00160: 1.22 vs. 0.91, *p* < 0.001; combined with BLLJ1626: 1.14 vs. 0.88, *p* = 0.02). Despite this apparent growth advantage in vitro, the actual proportion of secretory LnbX-positive *B. longum* in an infant population was shown to be only 0.2% of the *B. longum* total [82,83]. This corresponds with the lower percentage of infant-type gltA combined with lnbX. Furthermore, *B. longum* prioritizes many other sugars such as Gal, GlcNAc, and lactose over GNB or LNB utilization, which suggests that most infant gut *B. longum* strains prefer transporters for assimilating mainly LNT and prefer simple sugars over LNB. In this context, it is noteworthy that all strains encoded a complementary ABC transporter specific for GNB but not LNB (BLNG_00933-936) [31]. The higher prevalence rate of lnbX+ in combination with BLLJ_1626 might exist to compensate for the poor binding affinity for LNT of the latter. Nevertheless, BLLJ_1626+/lnbX- strains still grew to comparable OD_600_ values as other lnbX- strains, in contrast to the observation made by Yamada et al. [82], who reported the elimination of growth on LNT after the disruption of lnbX. We have no explanation for this discrepancy, although in the majority of strains, lnbX does not seem indispensable for growth on LNT. Our results, focusing on the HMO-degrading machinery, are in line with previous observations of a higher genetic diversity among *B. longum* strains compared to other *Bifidobacterium* species, except for *B. adolescentis* [15,58,59,60]. In the particular case of *B. longum* strains, their diversity and capabilities to metabolize a large range of carbohydrates have been suggested to arise from the influence of the intra-individual environment on epigenetic mechanisms, resulting in differential growth rates on carbohydrate substrates as adaptations to dietary changes during the early life developmental window [14], making them particularly powerful microbial competitors in the dynamic and complex human gut environment. We have shown that this diversity extends to the HMO-degrading machinery, although the phenotype was relatively stable due to the presence of complementary or redundant pathways for the degradation of LNT. Despite the finding that the presence of extracellular lnbX resulted in higher growth rates in vitro, thereby indicating a potential competitive feature, the percentage of lnbX-positive strains studied earlier seems relatively low at only 38% [49] compared to 64% in this study. However, it is likely that the “selfish” transporter-dependent intracellular digestion strategy enables the bifidobacteria to efficiently capture preferred carbon sources in the competitive ecosystem, providing an advantage over other gut microbes.

#### 3.3.4. *B. bifidum*

##### Results

**Figure 4:**
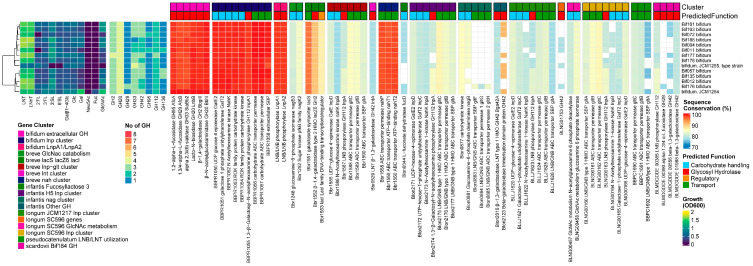
*B. bifidum*, from left to right: heatmap of growth on HMOs and building blocks (OD_600_); number of predicted GH genes and prevalence and sequence conservation rates of HMO utilization genes. The query gene clusters and predicted global function are color-coded on top, while the locus tag and protein name are shown at the bottom.

The growth on all individual HMOs was generally good for the *B. bifidum* strains, with better growth on 3′SL than 6′SL. However, a notable exception was the lower growth rates of JCM1255^T^ on all HMOs and the lack of growth on 3/6′SL. Additionally, two other strains did not grow on 2′FL. In contrast to most other human *Bifidobacterium* species, *B. bifidum* (and lnbX+ *B. longum*) strains use membrane-associated extracellular GHs to liberate mono- or di-saccharides from HMOs outside the cell. These are subsequently imported and further degraded in the cytoplasm. All 14 tested strains encoded the essential *B. bifidum*-specific extracellular GHs GH95 1,2-α-l-fucosidase (AfcA) [36], GH29 1,3/4-α-l-fucosidase (AfcB) [43], GH33 2,3/6-α-sialidase (SiaBb2) [46], GH2 β-1,4-galactosidase III (BbgIII), GH20 β-1,3-*N*-Acetylglucosaminidase I (BbhI) [44], GH20 Lacto-*N*-biosidase (LnbB) [41], GH112 (LnpA1) [64], and BbgIII at a very high homology rate (99.1% +/− 0.6%). Additionally, the prevalence of all genes encoded by the *lnp* cluster was 100%, and all genes were also highly conserved. We also found homologs for all genes from the *Bifidobacterium* nagR regulon, in concordance with the high conservation rates of other HMO-degrading machinery. Although *B. bifidum* does not seem to import large HMO structures, all strains seem to encode a full complement of genes to degrade LN(n)T intracellularly. We found a homolog to GH42 lntA/Bga42a (Blon_2016/Bbr_0529) LNT-β-1,3-galactosidase and GH2 β-1,4-galactosidase lacZ6 (Bbr_1552), as well as the fourth GH20 gene from *B. bifidum*. Interestingly, strain Bif176 apparently encoded a homolog to the gltFGH transporter from the *B. infantis nag* cluster and a homolog to the GH29 α-1-3/4-fucosidase that was unique to *B. scardovii* (Figure 4).

##### Discussion

In contrast to most other human *Bifidobacterium* species, *B. bifidum* (and lnbX+ *B. longum* strains) strains use membrane-associated extracellular GHs to liberate mono- or di-saccharides from HMOs outside the cell. These are subsequently imported and further degraded in the cytoplasm. As *B. bifidum* employs an external HMO-degrading strategy, six extracellular membrane-bound enzymes, one transporter, and one intracellular phosphorylase are essential for the assimilation of HMOs in this species. GH95 1,2-α-l-fucosidase (AfcA) [36], GH29 1,3/4-α-l-fucosidase (AfcB) [43], and GH33 2,3/6-α-sialidase (SiaBb2) [46] remove fucose and Neu5Ac units. Type I structures can be degraded in tandem by GH2 β-1,4-galactosidase III (BbgIII) and GH20 β-1,3-*N*-Acetylglucosaminidase I (BbhI) [44], or alternatively hydrolyzed into LNB and Lac by GH20 Lacto-*N*-biosidase (LnbB) [41]. The latter pathway can also be used to degrade type I structures. Free LNB is then imported (gltA) and subsequently phosphorolysed (GH112 LnpA1), releasing Gal-1-P,GlcNAc/GlcNAc-6-P [64] through the modified Leloir pathway encoded by the *lnp* cluster. Lac is either hydrolyzed by BbgIII or imported by Lac permease [76].

All 14 tested strains encoded the aforementioned essential genes at very high homology rates. In accordance with the genetic conservation, the degradation of all tested HMOs was relatively homogeneous. The growth on all individual HMOs was good, with better growth on 3′SL than 6′SL, in concordance with the preference of 2,3 over 3,6-linkages by SiaBb2 [46]. Nevertheless, a notable exception was the lack of growth on 3/6′SL by JCM1255^T^, although an absence of exo-α-sialidase activity has been reported for this strain previously [46]. This strains also contains a 17nt frameshift mutation in lnpA [84], which has been suggested to account for the limited growth of the strain on HMOs [85]. Indeed, we did observe significantly poorer growth on all other individual HMOs by this strain. Additionally, two other strains did not grow on 2′FL.

We observed the same distinctly high genetic conservation rate of all other genes encoded by the *lnp* cluster. However, one permease subunit of the ABC transporter (gltB, BBPR_1056) has been shown to be frequently absent (6 out of 10 strains) [48,86], and this genotype was assumed to be associated with reduced growth on mucin [48]. However, all 14 strains tested here, including JCM1255^T^, encoded BBPR_1056. Moreover, in these 2 studies, BBPR_1056 was shown to be both absent [48] and present [86] in JCM1255^T^. We currently do not have an explanation for the significant discrepancies in the conservation rates between these datasets. Notably, it seems unlikely this gene is sensitive to deletion in different working stocks of the type strain, considering the high conservation rates of all other genes involved in host glycan degradation.

We found homologs for all genes from the nagR regulon, which has recently been identified in most human bifidobacteria in the *lnp* and *nag* (nagK, nagB and nagA) clusters, in concordance with the high conservation rates of other HMO-degrading machinery. However, due to the specific extracellular strategy of *B. bifidum*, nagR also regulates multiple genes encoding GHs participating in extracellular HMO (LnbB, LnbX, BbgIII) and mucin O-glycan degradation in strain PRL2010. This transcription factor might, therefore, function as a global regulator of both HMO and mucin-*O*-glycan degradation in this species [65].

Interesting is the presence of lnbX in this regulon. Although all strains contained a homolog to *B. longum* GH136 lacto-*N*-biosidase LnbX and its chaperone LnbY (<50% coverage, 50% identity threshold) and both proteins are expressed in the cell, the gene is thought to be non-functional in *B. bifidum*, due to missing signal peptides in either LnbY or LnbX [80].

Although *B. bifidum* does not seem to import larger HMO structures, all strains seem to encode a full complement of genes to degrade LN(n)T intracellularly, including a homolog to the GH42 lntA/Bga42a (Blon_2016/Bbr_0529) LNT-β-1,3-galactosidase and GH2 β-1,4-galactosidase lacZ6 (Bbr_1552), as well as the fourth GH20 gene from *B. bifidum*. An intracellular GH20 β-N-acetylhexosaminidase (bbhIII) has shown activity towards para-nitrophenyl-β-linked-GlcNAc, although its biological meaning remains to be elucidated [44].

Interestingly, strain Bif176 apparently encoded a homolog to the gltFGH transporter from the *B. infantis nag* cluster that bound to LNB/GNB/Lewis a and type 1 H-trisaccharides (present in glycolipids and glycoproteins in several cell types, including the intestinal epithelium) [87], as well as a homolog to the GH29 α-1-3/4-fucosidase that was unique to *B. scardovii*. This genotypical feature may be interesting but apparently it did not lead to discernable changes in HMO utilization within our setup, although it might confer benefits in the competitive environment of the infant gut.

In conclusion, all surveyed genes showed very high prevalence and conservation rates in this species, which were mirrored by a relatively homogeneous HMO degradation phenotype. However, in contrast to other species, very small mutations have been shown to lead to large phenotypical differences, especially for the type strain JCM1255^T^, which has a 17nt frameshift mutation in lnpA that (potentially) interferes with LNB utilization. We also observed the significantly lower growth rates of this strain on substrates that produce LNB as intermediate steps during their catabolism. There might also be mutations in its siaBb2, which completely lacks exo-α-sialidase activity [46], although all genes in our set were highly alike, similarly to the GH95 1,2-α-l-fucosidase (AfcA) in strains Bif072 and Bif011, which showed no growth on 2‘FL. The main distinction between *B. bifidum* and other bifidobacteria is its capacity to grow on both HMOs and mucin. This seemingly unique capability, facilitated by its large repertoire of extracellular GHs, may have led to the lack of loss or acquisition of other carbohydrate-metabolizing abilities, suggesting this capability provides a strong selective advantage over other (bifido)bacteria [86].

#### 3.3.5. *B. pseudocatenulatum*

##### Results

All *B. pseudocatenulatum* strains grew on LNT only, with one strain growing well on 2′FL and 3FL. We found homologs for both the prototypical β-1,3/1,4-galactosidases Bga42A/lntA (Blon_2016/Bbr_0529) and Bga2A (blon_2334) in all strains and also identified a GH20 β-*N*-acetylhexoaminidase. Therefore, all five tested strains contained at least one member of the required GH families for LN(n)T degradation (GH2, GH42, GH20). The GH20 β-*N*-acetylhexoaminidase displayed no significant homology to any of the ‘main’ *Bifidobacterium* GH20 β-N-acetylhexoaminidases, and its presence was restricted to the species *B. pseudocatenulatum* and *B. catenulatum*.

**Figure 5:**
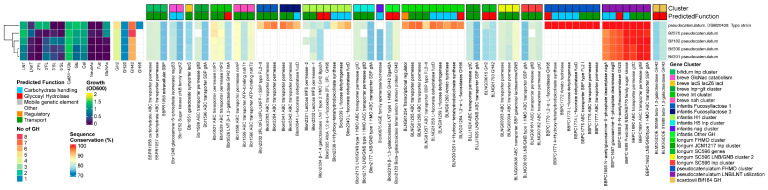
*B. pseudocatenulatum*, from left to right: heatmap of growth on HMOs and building blocks (OD_600_); number of predicted GH genes and prevalence and sequence conservation rates of HMO utilization genes. The query gene clusters and predicted global function are color-coded on top, while the locus tag and protein name are shown at the bottom.

With regards to the transporters, we identified homologs to structural units of the GNB/galactosyl/lactosamine transporter from *B. longum* (BLNG_0933-0936, missing the ATP-binding module) and of both *B. breve nah* and *lnt* transporters, missing both SBPs for the uptake of LN(n)T and LNT, respectively. In contrast, *B. pseudocatenulatum* contained the gltABC transporter, which was combined with LNB/LNT utilization machinery (nagA, nagB, and the GH20 β-*N*-acetylhexoaminidase). Instead of the conventional *lnp* cluster layout that combines this transporter with the modified Leloir-like pathway for processing LNB/GNB, type I HMOs and glycolipids, and intestinal glycans, like the other infant bifidobacteria [38,73,88], we additionally found a homolog to the nagK from *B. breve* outside this cluster. We confirmed the presence of a complete FHMO cluster in the type strain DSM20438^T^, which contains a GH95 α-1,2 fucosidase, which was more similar to genes from other FHMO clusters [17,21,50] than Blon_2335/AfcA in H1 from *B. infantis* (Figure 5).

##### Discussion

Unlike other *Bifidobacterium* species more common to breastfed infants such as *B. infantis*, *B. longum*, *B. breve*, or *B. bifidum*, *B. pseudocatenulatum* is underexamined, despite its frequent presence in breastfed infant feces [12,18,19,20,21], although it is also prevalent in adults [12,16,19,89]. All 6 tested strains contained at least one member of the required GH families for LN(n)T degradation (GH2, GH42, GH20), although the 100% prevalence of Bga42A/lntA in this strain set is in contrast to the ~30% previously reported for 64 publicly available genomes [49]. The *B. pseudocatenulatum-* and *B. catenulatum*-specific GH20 β-*N*-acetylhexoaminidase displayed no significant homology to any of the ‘main’ *Bifidobacterium* GH20 β-*N*-acetylhexoaminidases, although we can assume its function due to the growth of all strains of LNT.

For the uptake of LNT, strains of this species use the *B. pseudocatenulatum-* and *B. catenulatum*-specific LNB/LNT utilization cluster (gltABC, nagA, nagB, and the β-*N*-acetylhexoaminidase, combined with nagK and bga42A), which is under influence of the nagR regulon, resulting in the de-repression of nagR-controlled genes due to the release of GlcNac by the activity of the GH20 β-*N*-acetylhexoaminidase [46], thereby explaining the upregulation of the LNB/LNT utilization cluster by LNFPI but not 2′FL [19]. This machinery explains the growth of all strains on LNT, which is in concordance with previous work [19]. Nevertheless, one study reported minimal growth on LNnT for one out of 10 infant isolates [18], which might have been non-specific growth.

Additionally, we confirmed the presence of a complete FHMO cluster in the type strain DSM20438^T^ [19]. The catalytic specificity rates of GH95 α-fucosidases differ among *Bifidobacterium* species, and this 1,2-α-l-fucosidase has shown cross-reactivity on 1,3-linkages in 3FL, as strains of *B. pseudocatenulatum* and *B. breve* grew well on both 2′FL and 3FL without a dedicated GH29 α-1,3/4 fucosidase [19]. This explains the growth of this strain on both 2′FL and 3FL, although it is only equipped with a GH95 α-fucosidase. Nevertheless, the presence of a GH29 α-1,3/4-fucosidase in this cluster [19] has been shown to promote the cleavage of an additional range of HMO moieties that are poorly cleaved by the GH95 enzyme, suggesting that the addition of the second α-fucosidase expands the pool of fucosylated HMOs that are catabolized. Therefore, similar to other species, although growth experiments with a limited range of HMOs show comparable growth characteristics, the substrate competition in vivo may still show a growth advantage to strains possessing the complementary GH29 α-1,3/4-fucosidase [19].

The prevalence of these complete FHMO utilization loci in *B. pseudocatenulatum* is estimated to be low (<10%). However, this might be strongly influenced by their origin, as no α-fucosidase-positive strains were identified in 45 strains from Vietnam (isolated from children and adults), while in two 3–5-month-old exclusively breastfed, vaginally born UK infants, 1 infant contained a GH95+ strain. Similarly, 3 out of 6 strains isolated from Japanese vaginally born breastfed infants [21] contained the complete FHMO locus [21], suggesting a strong association of FHMO+ strains with infant ecosystems. Nevertheless, surprisingly for *B. longum*, strains capable of metabolizing FHMOs were also isolated from a formula-fed baby that only received standard non-supplemented (i.e., no prebiotics or synthetic HMOs) formula [14].

The fact that *B. pseudocatenulatum* seems to have an open pan-genome, approaching saturation [60], combined with overrepresentation of the FHMO cluster in infants and the presence of *B. pseudocatenulatum* in adults, suggests genomic flexibility and is indicative of (rapid) adaptation to resource availability. The pan-genome of *B. pseudocatenulatum* may expand mainly with genes responsible for carbohydrate transport and metabolism through horizontal gene transfer (HGT) with other *Bifidobacterium* species, thereby expediting the diversification of clonal bacteria. *B. pseudocatenulatum* populations were shown to undergo a prolonged period of within-host evolution and expansion, leading to a strain-level differential response to a dietary intervention due to differences between strains in gene copy numbers of carbohydrate-active enzymes targeting plant polysaccharides [90].

#### 3.3.6. *B. catenulatum*

##### Results

**Figure 6:**
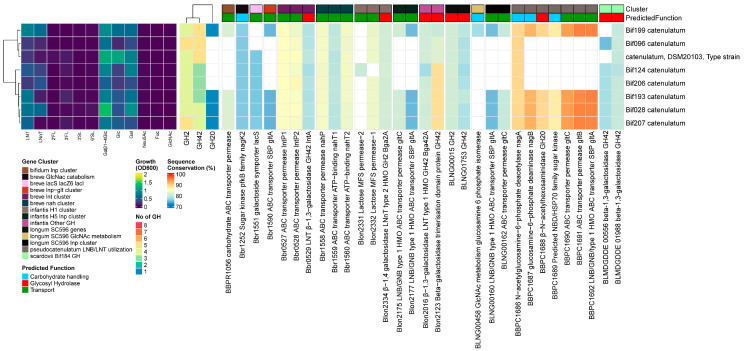
*B. catenulatum*, from left to right: heatmap of growth on HMOs and building blocks (OD_600_); number of predicted GH genes and prevalence and sequence conservation rates of HMO utilization genes. The query gene clusters and predicted global function are color-coded on top, while the locus tag and protein name are shown at the bottom.

Four out of 8 *B. catenulatum* strains grew moderately on LNT. In all strains, we found homologs for both prototypical β-1,3/1,4-galactosidases, Bga42A/lntA (Blon_2016/Bbr_0529) and Bga2A (Blon_2334). Four strains additionally encoded a GH20 β-N-acetylhexoaminidase related to the *B. pseudocatenulatum* gene BBPC_1688. In these strains, we also identified homologs to the same complement of transporters as *B. pseudocatenulatum*, except for BLNG_0933-0935. We also found the LNB/LNT utilization cluster and other genes under the NagR regulon influence [65], as described previously for *B. pseudocatenulatum*. However, the genes in this cluster are slightly different, while the gltABC transporter has slightly higher similarity rates. Strains with and without the LNB/LNT utilization cluster grew to OD_600_ 0.70 ± 0.15 vs. 0.32 ± 0.14 (*p* < 0.001) on LNT, respectively (Figure 6).

##### Discussion

The presence of a full complement of GHs (GH2, GH42, GH20) for the internal sequential degradation of LN(n)T, combined with the LNB/LNT utilization cluster and other genes under the NagR regulon influence [65], as described above for *B. pseudocatenulatum*, explains the growth on LNT by strains containing the LNB/LNT utilization cluster, similar to *B. pseudocatenulatum*. *B. catenulatum* consists of the subspecies *kashiwanohense* and *catenulatum*, of which the latter is mainly found in adults [61,89,91] but also infants [61] and was shown to be shared between an infant–mother pair [92]. Previous comparative genomic analyses between the two subspecies *kashiwanohense* and *catenulatum* identified the FHMO cluster in all (5) *kashiwanohense* strains but not (yet) in *B. catenulatum* (11 strains, including 2 infant isolates). Additionally, the GH reservoir of *B. catenulatum* was more directed to plant-derived glycans present in the adult intestine. Only 1 infant and 1 adult-derived strain encoded a GH20 gene, giving them the full complement of GHs for the internal sequential degradation of LN(n)T [61], which is in contrast to our findings, where 4 out of 8 strains encoded a GH20 gene. As far as we know, this is the first set of *B. catenulatum* strains to be tested for growth on HMO, and our results suggest that the infant type *B. catenulatum* strains encode and express genes closely related to the *B. pseudocatenulatum* LNB/LNT utilization cluster, enabling the internalization and degradation of LNT, while the adult type has lost this ability, which provides a rationale for the presence and prevalence of this species in both infants and adults. Additionally, although the number of strains was low, the pan-genome of *B. catenulatum* tended to be more open than *B. kashiwanohense*, suggesting more flexible environmental adaptability for the former. In contrast, *kashiwanohense* is specialized to infants, with 100% prevalence of the FHMO cluster so far [61], while this has not been identified yet in *B. catenulatum*.

#### 3.3.7. *B. adolescentis*

##### Results

**Figure 7:**
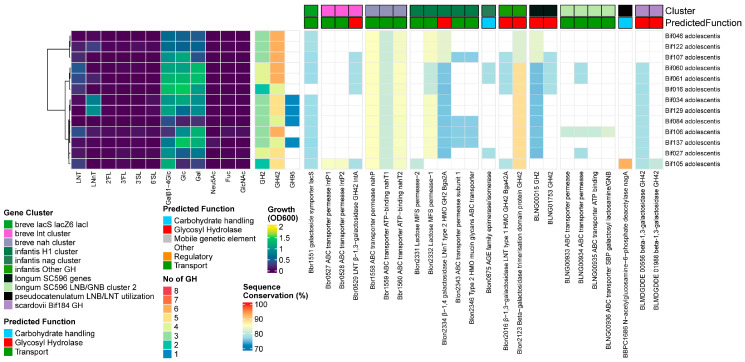
*B. adolescentis*, from left to right: heatmap of growth on HMOs and building blocks (OD_600_); number of predicted GH genes and prevalence and sequence conservation rates of HMO utilization genes. The query gene clusters and predicted global function are color-coded on top, while the locus tag and protein name are shown at the bottom.

None of the 13 *B. adolescentis* strains grew on HMOs, except for Bif034 and Bif129 on LNnT. We found homologs for both prototypical *Bifidobacterium* HMO-degrading β-1,4/1,3-galactosidases, although their presence was infrequent (12/13 for Bga2A (Blon_2334) and 5/13 Bga42A/lntA (Blon_2016/Bbr_0529)), contrary to all other species tested. None of the strains encoded α-sialidases, while four of the strains contained a GH95 α-fucosidase. With regards to the transporters, we found homologs to the complete GNB/galactosyl/lactosamine transporter from *B. longum* (BLNG_0933-0936) and structural units of the *B. breve* nah transporter, although nahS (the LN(n)T SBP) was missing. No homologs to any *lnp* clusters or the *B. pseudocatenulatum–B. catenulatum* LNB/GNB/LNT catabolic cluster was found, nor were genes under the influence of the NagR regulon, as found in HMO-utilizing bifidobacteria [65] (Figure 7).

##### Discussion

This explains why none of the strain grew on any of the tested HMOs, except for Bif034 and Bif129 on LNnT. One strain (Bif106) showed potential towards host-derived glycans due to the presence of (a combination of) the *B. longum* transporter (BLNG_0933-0936) and a homolog of Bga42A/lntA (Blon_2016/Bbr_0529). However, this was not a strain that grew on LNnT, and we currently have no explanation for the latter. These observations largely support a pan-genomic analysis of several *B. adolescentis* genomes, showing that this species has specialized itself towards the utilization of plant-derived glycans, which are normally present with high abundance in adult diets [59].

#### 3.3.8. *B. scardovii*

##### Results

**Figure 8:**
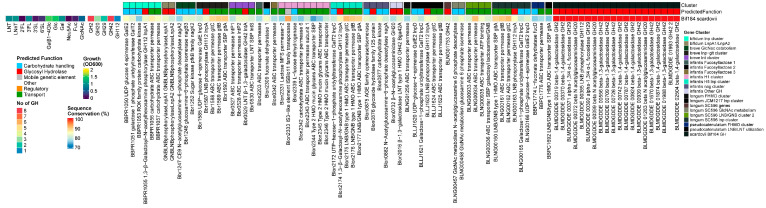
*B. scardovii*, from left to right: heatmap of growth on HMOs and building blocks (OD_600_); number of predicted GH genes and prevalence and sequence conservation rates of HMO utilization genes. The query gene clusters and predicted global function are color-coded on top, while the locus tag and protein name are shown at the bottom.

The *B. scardovii* strain grew well on LN(n)T and 3FL. We found homologs to the GH42 β-1,3-galactosidase Bga42A/lntA (Blon_2016/Bbr_0529) and GH20 β-*N*-acetylhexoaminidase (Blon_0732) but not to the prototypical GH2 β-1,4-galactosidase Bga2A/lacZ6 (Blon_2334/Bbr_1552). Nevertheless, we predicted 6 other GH2 enzymes that were unique for this species, as well as 2 unique GH29 genes. With regards to the transporters, we identified homologs to a partial *B. breve* lnt cluster (missing lntR), the complete GNB/galactosyl/lactosamine transporter from *B. longum* (BLNG_0933-0936), and a partial *lnp* cluster (missing nahK/lnpB). Interestingly, it contains the type II HMO/mucin glycan transporter, similar to the *B. infantis* H1 cluster hmoA2B2C2 (Blon_2342-4346) [65], which was not found in the *B. scardovii* type strain [65]. Strikingly, it is the only HMO-degrading strain without a homolog to the *B. breve nah* transporter. The genome also contained a homolog to the partial *nag* cluster of nagA, nagB, and nagK, giving it a similar nagR regulon structure as *B. longum*, with hmoA2B2C2 potentially included as well. It also contained homologs to several fucose catabolism genes from *B. infantis* H1, namely fucD, one ABC transporter permease subunit, and an L-fucose dehydrogenase (Figure 8).

##### Discussion

This genetic machinery explains the good growth of this strain on LN(n)T because Bga42A/lntA has cross-reactivity towards β-1,4-linkages in LNnT. Most bifidobacteria have a dedicated GH2 β-1,4-galactosidase, and one of the predicted GH2 enzymes unique for this species had no known substrate, meaning it might still encode a specialist enzyme with activity towards the terminal Gal in type II chains. However, we cannot explain the growth on 3FL. Despite the genome encoding the necessary GH29 fucosidases and homologs to several fucose catabolism genes, we did not identify a FL transporter. Although *Bifidobacterium* species are frequently isolated from the human intestine, the type strain JCM 12489^T^ was isolated from human blood. *B. scardovii* can use LNB as a supplementary carbon source [93], although as far as we know, this is the first strain for which growth on HMOs has been tested.

## 4. Conclusions

In conclusion, our findings highlight the extent of the genetic diversity between strains of the same species and demonstrate the effects of strain-level genetic diversity on species-specific HMO degradation pathways and utilization. Most strains retained their species-specific HMO degradation profile through the conservation of a core set of genes or through the presence of overlapping or redundant genes or pathways. In particular, the *B. infantis* strains showed large differences in their configuration of complete HMO utilization loci to potentially alter their HMO utilization preference via the gene deletion of transporters to restrict the range of internalized HMOs, while retaining most of the functionality through genetic redundancy. These different types of genetic diversity within species and the specialization of species, although not visible with growth on the individual HMOs only measured at one timepoint as in this study, likely influence competitive behavior for HMO foraging in situ between different *Bifidobacterium* strains and species.

Additionally, the presence of a partial FHMO cluster in all analyzed *B. breve* strains, in contrast to its complete presence or absence in other datasets, combined with one strain containing an additional GH29 α-1-3/4-fucosidase, hints at the potential of *B. breve* strains with wider HMO assimilation phenotypes that might still be discovered. Further, we complement the little data available for *B. catenulatum* and *B. scardovii* by showing that some strains of the former grow on LNT and some of the latter on LN(n)T and 3FL by identifying the utilized gene sets. We also found that *B. longum* strains encoded two different versions of the *lnp* cluster in combination with lnbX and determined the effect of this genotype on the growth on LNT.

Exploring the genomic diversity of strains across different species will help to refine our understanding of the genetic factors that contribute to the persistence of individual strains in different ecological contexts, such as the ability to utilize a specific set of HMOs and the formation of cross-feeding networks with other (*Bifidobacterium*) species. The current methods of microbiome research generally lack the resolution to discern strain-specific differences that shape the complex network of host–microbe interactions in the human gut. Even within the simplest example, the infant gut, where the microbiome’s complexity is a fraction of that in an adult, the impact of strain-specific metabolic variations on this ecosystem is not well understood [17,27,33,69]. Defining individual strain’s roles within the complex system will be essential for understanding the numerous interactions affecting host health throughout life, and could be used as a guide for health-promoting supplementation. Therefore, detailed mechanistic knowledge of the relationships between substrates and specific strains within a species will inform effective and rationalized prebiotic and probiotic strategies. Single or multi-strain synbiotic supplements could be designed to pair specific HMOs with specific strains that will consume them via known strategies to achieve predefined functional outcomes and for selective stimulation in a competitive environment.

## Data Availability

The raw data supporting the conclusions of this article will be made available by the authors on request. The data are not publicly available because they are part of ongoing research.

## Supplementary Materials

The following supporting information can be downloaded at https://www.mdpi.com/article/10.3390/nu16121893/s1: Figure S1. Heatmap of growth on HMOs, number of predicted GH genes, and prevalence and sequence conservation of HMO utilization genes in all strains used in this study. Figure S2. Heatmap of growth on HMOs, number of predicted GH genes, and prevalence and sequence conservation of HMO utilization genes from *B. animalis*. Figure S3. Heatmap of growth on HMOs, number of predicted GH genes, and prevalence and sequence conservation of HMO utilization genes from *B. angulatum*. Table S1. Used query nucleotide sequences and accession numbers. Table S2. Sequencing statistics and genomic features of the Bifidobacterium strains used in this study. Table S3. Species-specific genome sizes (nt). Table S4. Species-specific genomic GC content (%). Table S5. Species-specific number of ORFs.

## References

[1] Wampach L., Heintz-Buschart A., Fritz J.V., Ramiro-Garcia J., Habier J., Herold M., Narayanasamy S., Kaysen A., Hogan A.H., Bindl L. (2018). Birth Mode Is Associated with Earliest Strain-Conferred Gut Microbiome Functions and Immunostimulatory Potential. Nat. Commun..

[2] Tamburini S., Shen N., Wu H.C., Clemente J.C. (2016). The Microbiome in Early Life: Implications for Health Outcomes. Nat. Med..

[3] UNICEF, WHO (2018). Capture the Moment—Early Initiation of Breastfeeding: The Best Start for Every Newborn.

[4] Bokulich N.A., Chung J., Battaglia T., Henderson N., Jay M., Li H., Lieber A.D., Wu F., Perez-Perez G.I., Chen Y. (2016). Antibiotics, Birth Mode, and Diet Shape Microbiome Maturation during Early Life. Sci. Transl. Med..

[5] Hermes G.D.A., Eckermann H.A., de Vos W.M., de Weerth C. (2020). Does Entry to Center-Based Childcare Affect Gut Microbial Colonization in Young Infants?. Sci. Rep..

[6] Van Daele E., Kamphorst K., Vlieger A.M., Hermes G., Milani C., Ventura M., Belzer C., Smidt H., van Elburg R.M., Knol J. (2022). Effect of Antibiotics in the First Week of Life on Faecal Microbiota Development. Arch. Dis. Child. Fetal Neonatal Ed..

[7] Rothschild D., Weissbrod O., Barkan E., Kurilshikov A., Korem T., Zeevi D., Costea P.I., Godneva A., Kalka I.N., Bar N. (2018). Environment Dominates over Host Genetics in Shaping Human Gut Microbiota. Nature.

[8] Bogaert D., van Beveren G.J., de Koff E.M., Lusarreta Parga P., Balcazar Lopez C.E., Koppensteiner L., Clerc M., Hasrat R., Arp K., Chu M.L.J.N. (2023). Mother-to-Infant Microbiota Transmission and Infant Microbiota Development across Multiple Body Sites. Cell Host Microbe.

[9] Melsaether C., Høtoft D., Wellejus A., Hermes G.D.A., Damholt A. (2023). Seeding the Infant Gut in Early Life-Effects of Maternal and Infant Seeding with Probiotics on Strain Transfer, Microbiota, and Gastrointestinal Symptoms in Healthy Breastfed Infants. Nutrients.

[10] De Agüero M.G., Ganal-Vonarburg S.C., Fuhrer T., Rupp S., Uchimura Y., Li H., Steinert A., Heikenwalder M., Hapfelmeier S., Sauer U. (2016). The Maternal Microbiota Drives Early Postnatal Innate Immune Development. Science.

[11] Ventura M., Canchaya C., Tauch A., Chandra G., Fitzgerald G.F., Chater K.F., van Sinderen D. (2007). Genomics of Actinobacteria: Tracing the Evolutionary History of an Ancient Phylum. Microbiol. Mol. Biol. Rev..

[12] Turroni F., Foroni E., Pizzetti P., Giubellini V., Ribbera A., Merusi P., Cagnasso P., Bizzarri B., De’Angelis G.L., Shanahan F. (2009). Exploring the Diversity of the Bifidobacterial Population in the Human Intestinal Tract. Appl. Environ. Microbiol..

[13] Avershina E., Storrø O., Øien T., Johnsen R., Wilson R., Egeland T., Rudia K. (2013). Bifidobacterial Succession and Correlation Networks in a Large Unselected Cohort of Mothers and Their Children. Appl. Environ. Microbiol..

[14] Kujawska M., La Rosa S.L., Roger L.C., Pope P.B., Hoyles L., McCartney A.L., Hall L.J. (2020). Succession of Bifidobacterium Longum Strains in Response to a Changing Early Life Nutritional Environment Reveals Dietary Substrate Adaptations. iScience.

[15] Arboleya S., Bottacini F., O’Connell-Motherway M., Ryan C.A., Ross R.P., van Sinderen D., Stanton C. (2018). Gene-Trait Matching across the Bifidobacterium Longum Pan-Genome Reveals Considerable Diversity in Carbohydrate Catabolism among Human Infant Strains. BMC Genom..

[16] Chung The H., Nguyen Ngoc Minh C., Tran Thi Hong C., Nguyen Thi Nguyen T., Pike L.J., Zellmer C., Pham Duc T., Tran T.-A., Ha Thanh T., Van M.P. (2021). Exploring the Genomic Diversity and Antimicrobial Susceptibility of Bifidobacterium Pseudocatenulatum in a Vietnamese Population. Microbiol. Spectr..

[17] Bunesova V., Lacroix C., Schwab C. (2016). Fucosyllactose and L-Fucose Utilization of Infant Bifidobacterium Longum and Bifidobacterium Kashiwanohense. BMC Microbiol..

[18] Lawson M.A.E., O’Neill I.J., Kujawska M., Gowrinadh Javvadi S., Wijeyesekera A., Flegg Z., Chalklen L., Hall L.J. (2019). Breast Milk-Derived Human Milk Oligosaccharides Promote Bifidobacterium Interactions within a Single Ecosystem. ISME J..

[19] Shani G., Hoeflinger J.L., Heiss B.E., Masarweh C.F., Larke J.A., Jensen N.M., Wickramasinghe S., Davis J.C., Goonatilleke E., El-Hawiet A. (2022). Fucosylated Human Milk Oligosaccharide Foraging within the Species Bifidobacterium Pseudocatenulatum Is Driven by Glycosyl Hydrolase Content and Specificity. Appl. Environ. Microbiol..

[20] Ruiz-Moyano S., Totten S.M., Garrido D.A., Smilowitz J.T., Bruce German J., Lebrilla C.B., Mills D.A. (2013). Variation in Consumption of Human Milk Oligosaccharides by Infant Gut-Associated Strains of Bifidobacterium Breve. Appl. Environ. Microbiol..

[21] Matsuki T., Yahagi K., Mori H., Matsumoto H., Hara T., Tajima S., Ogawa E., Kodama H., Yamamoto K., Yamada T. (2016). A Key Genetic Factor for Fucosyllactose Utilization Affects Infant Gut Microbiota Development. Nat. Commun..

[22] Engfer M.B., Stahl B., Finke B., Sawatzki G., Daniel H. (2000). Human Milk Oligosaccharides Are Resistant to Enzymatic Hydrolysis in the Upper Gastrointestinal Tract. Am. J. Clin. Nutr..

[23] Parschat K., Melsaether C., Jäpelt K.R., Jennewein S. (2021). Clinical Evaluation of 16-Week Supplementation with 5HMO-Mix in Healthy-Term Human Infants to Determine Tolerability, Safety, and Effect on Growth. Nutrients.

[24] Erney R.M., Malone W.T., Skelding M.B., Marcon A.A., Kleman–Leyer K.M., O’Ryan M.L., Ruiz–Palacios G., Hilty M.D., Pickering L.K., Prieto P.A. (2000). Variability of Human Milk Neutral Oligosaccharides in a Diverse Population. J. Pediatr. Gastroenterol. Nutr..

[25] McGuire M.K., Meehan C.L., McGuire M.A., Williams J.E., Foster J., Sellen D.W., Kamau-Mbuthia E.W., Kamundia E.W., Mbugua S., Moore S.E. (2017). What’s Normal? Oligosaccharide Concentrations and Profiles in Milk Produced by Healthy Women Vary Geographically. Am. J. Clin. Nutr..

[26] Duar R.M., Casaburi G., Mitchell R.D., Scofield L.N.C., Ortega Ramirez C.A., Barile D., Henrick B.M., Frese S.A. (2020). Comparative Genome Analysis of Bifidobacterium Longum Subsp. Infantis Strains Reveals Variation in Human Milk Oligosaccharide Utilization Genes among Commercial Probiotics. Nutrients.

[27] Zabel B.E., Gerdes S., Evans K.C., Nedveck D., Singles S.K., Volk B., Budinoff C. (2020). Strain-Specific Strategies of 2′-Fucosyllactose, 3-Fucosyllactose, and Difucosyllactose Assimilation by Bifidobacterium Longum Subsp. Infantis Bi-26 and ATCC 15697. Sci. Rep..

[28] Sela D.A., Garrido D., Lerno L., Wu S., Tan K., Eom H.J., Joachimiak A., Lebrilla C.B., Mills D.A. (2012). Bifidobacterium Longum Subsp. Infantis ATCC 15697 α-Fucosidases Are Active on Fucosylated Human Milk Oligosaccharides. Appl. Environ. Microbiol..

[29] Ward R.E., Niñonuevo M., Mills D.A., Lebrilla C.B., German J.B. (2007). In Vitro Fermentability of Human Milk Oligosaccharides by Several Strains of Bifidobacteria. Mol. Nutr. Food Res..

[30] Garrido D., Ruiz-Moyano S., Lemay D.G., Sela D.A., German J.B., Mills D.A. (2015). Comparative Transcriptomics Reveals Key Differences in the Response to Milk Oligosaccharides of Infant Gut-Associated Bifidobacteria. Sci. Rep..

[31] Garrido D., Ruiz-Moyano S., Kirmiz N., Davis J.C., Totten S.M., Lemay D.G., Ugalde J.A., German J.B., Lebrilla C.B., Mills D.A. (2016). A Novel Gene Cluster Allows Preferential Utilization of Fucosylated Milk Oligosaccharides in Bifidobacterium Longum Subsp. Longum SC596. Sci. Rep..

[32] James K., Motherway M.O.C., Bottacini F., Van Sinderen D. (2016). Bifidobacterium Breve UCC2003 Metabolises the Human Milk Oligosaccharides Lacto-N-Tetraose and Lacto-N-Neo-Tetraose through Overlapping, yet Distinct Pathways. Sci. Rep..

[33] Özcan E., Sela D.A. (2018). Inefficient Metabolism of the Human Milk Oligosaccharides Lacto-N-Tetraose and Lacto-N-Neotetraose Shifts Bifidobacterium Longum Subsp. Infantis Physiology. Front. Nutr..

[34] Nishiyama K., Nagai A., Uribayashi K., Yamamoto Y., Mukai T., Okada N. (2018). Two Extracellular Sialidases from Bifidobacterium Bifidum Promote the Degradation of Sialyl-Oligosaccharides and Support the Growth of Bifidobacterium Breve. Anaerobe.

[35] Wong C.B., Odamaki T., Xiao J.Z. (2020). Insights into the Reason of Human-Residential Bifidobacteria (HRB) Being the Natural Inhabitants of the Human Gut and Their Potential Health-Promoting Benefits. FEMS Microbiol. Rev..

[36] Katayama T., Sakuma A., Kimura T., Makimura Y., Hiratake J., Sakata K., Yamanoi T., Kumagai H., Yamamoto K. (2004). Molecular Cloning and Characterization of Bifidobacterium Bifidum 1,2-α-L-Fucosidase (AfcA), a Novel Inverting Glycosidase (Glycoside Hydrolase Family 95). J. Bacteriol..

[37] Yoshida E., Sakurama H., Kiyohara M., Nakajima M., Kitaoka M., Ashida H., Hirose J., Katayama T., Yamamoto K., Kumagai H. (2012). Bifidobacterium Longum Subsp. Infantis Uses Two Different β-Galactosidases for Selectively Degrading Type-1 and Type-2 Human Milk Oligosaccharides. Glycobiology.

[38] Kitaoka M., Tian J., Nishimoto M. (2005). Novel Putative Galactose Operon Involving Lacto-N-Biose Phosphorylase in Bifidobacterium Longum. Appl. Environ. Microbiol..

[39] Fujita K., Oura F., Nagamine N., Katayama T., Hiratake J., Sakata K., Kumagai H., Yamamoto K. (2005). Identification and Molecular Cloning of a Novel Glycoside Hydrolase Family of Core 1 Type O-Glycan-Specific Endo-Alpha-N-Acetylgalactosaminidase from Bifidobacterium Longum. J. Biol. Chem..

[40] Nagae M., Tsuchiya A., Katayama T., Yamamoto K., Wakatsuki S., Kato R. (2007). Structural Basis of the Catalytic Reaction Mechanism of Novel 1,2-Alpha-L-Fucosidase from Bifidobacterium Bifidum. J. Biol. Chem..

[41] Wada J., Ando T., Kiyohara M., Ashida H., Kitaoka M., Yamaguchi M., Kumagai H., Katayama T., Yamamoto K. (2008). Bifidobacterium Bifidum Lacto-N-Biosidase, a Critical Enzyme for the Degradation of Human Milk Oligosaccharides with a Type 1 Structure. Appl. Environ. Microbiol..

[42] Goulas T., Goulas A., Tzortzis G., Gibson G.R. (2009). Comparative Analysis of Four β-Galactosidases from Bifidobacterium Bifidum NCIMB41171: Purification and Biochemical Characterisation. Appl. Microbiol. Biotechnol..

[43] Ashida H., Miyake A., Kiyohara M., Wada J., Yoshida E., Kumagai H., Katayama T., Yamamoto K. (2009). Two Distinct α-L-Fucosidases from Bifidobacterium Bifidum Are Essential for the Utilization of Fucosylated Milk Oligosaccharides and Glycoconjugates. Glycobiology.

[44] Miwa M., Horimoto T., Kiyohara M., Katayama T., Kitaoka M., Ashida H., Yamamoto K. (2010). Cooperation of β-Galactosidase and β-N-Acetylhexosaminidase from Bifidobacteria in Assimilation of Human Milk Oligosaccharides with Type 2 Structure. Glycobiology.

[45] Garrido D., Ruiz-Moyano S., Mills D.A. (2012). Release and Utilization of N-Acetyl-d-Glucosamine from Human Milk Oligosaccharides by Bifidobacterium Longum Subsp. Infantis. Anaerobe.

[46] Kiyohara M., Tanigawa K., Chaiwangsri T., Katayama T., Ashida H., Yamamoto K. (2011). An Exo—Sialidase from Bifidobacteria Involved in the Degradation of Sialyloligosaccharides in Human Milk and Intestinal Glycoconjugates. Glycobiology.

[47] Hidaka M., Nishimoto M., Kitaoka M., Wakagi T., Shoun H., Fushinobu S. (2009). The Crystal Structure of Galacto-N-Biose/Lacto-N-Biose I Phosphorylase. A Large Deformation of a Tim Barrel Scaffold. J. Biol. Chem..

[48] Turroni F., Bottacini F., Foroni E., Mulder I., Kim J.H., Zomer A., Sánchez B., Bidossi A., Ferrarini A., Giubellini V. (2010). Genome Analysis of Bifidobacterium Bifidum PRL2010 Reveals Metabolic Pathways for Host-Derived Glycan Foraging. Proc. Natl. Acad. Sci. USA.

[49] Sakanaka M., Gotoh A., Yoshida K., Odamaki T., Koguchi H., Xiao J.Z., Kitaoka M., Katayama T. (2020). Varied Pathways of Infant Gut-Associated Bifidobacterium to Assimilate Human Milk Oligosaccharides: Prevalence of the Gene Set and Its Correlation with Bifidobacteria-Rich Microbiota Formation. Nutrients.

[50] Garrido D., Kim J.H., German J.B., Raybould H.E., Mills D.A. (2011). Oligosaccharide Binding Proteins from Bifidobacterium Longum Subsp. Infantis Reveal a Preference for Host Glycans. PLoS ONE.

[51] Bankevich A., Nurk S., Antipov D., Gurevich A.A., Dvorkin M., Kulikov A.S., Lesin V.M., Nikolenko S.I., Pham S., Prjibelski A.D. (2012). SPAdes: A New Genome Assembly Algorithm and Its Applications to Single-Cell Sequencing. J. Comput. Biol..

[52] Seemann T. (2014). Prokka: Rapid Prokaryotic Genome Annotation. Bioinformatics.

[53] Chklovski A., Parks D.H., Woodcroft B.J., Tyson G.W. (2023). CheckM2: A Rapid, Scalable and Accurate Tool for Assessing Microbial Genome Quality Using Machine Learning. Nat. Methods.

[54] Drula E., Garron M.L., Dogan S., Lombard V., Henrissat B., Terrapon N. (2022). The Carbohydrate-Active Enzyme Database: Functions and Literature. Nucleic Acids Res..

[55] Bortolaia V., Kaas R.S., Ruppe E., Roberts M.C., Schwarz S., Cattoir V., Philippon A., Allesoe R.L., Rebelo A.R., Florensa A.F. (2020). ResFinder 4.0 for Predictions of Phenotypes from Genotypes. J. Antimicrob. Chemother..

[56] R Core Team (2022). R: A Language and Environment for Statistical Computing.

[57] Gu Z., Eils R., Schlesner M. (2016). Complex Heatmaps Reveal Patterns and Correlations in Multidimensional Genomic Data. Bioinformatics.

[58] Bottacini F., Morrissey R., Esteban-Torres M., James K., Van Breen J., Dikareva E., Egan M., Lambert J., Van Limpt K., Knol J. (2018). Comparative Genomics and Genotype-Phenotype Associations in Bifidobacterium Breve. Sci. Rep..

[59] Duranti S., Milani C., Lugli G.A., Mancabelli L., Turroni F., Ferrario C., Mangifesta M., Viappiani A., Sanchez B., Margolles A. (2016). Evaluation of Genetic Diversity among Strains of the Human Gut Commensal Bifidobacterium Adolescentis. Sci. Rep..

[60] Lin G., Liu Q., Wang L., Li H., Zhao J., Zhang H., Wang G., Chen W. (2022). The Comparative Analysis of Genomic Diversity and Genes Involved in Carbohydrate Metabolism of Eighty-Eight Bifidobacterium Pseudocatenulatum Isolates from Different Niches of China. Nutrients.

[61] Liu J., Li W., Yao C., Yu J., Zhang H. (2022). Comparative Genomic Analysis Revealed Genetic Divergence between Bifidobacterium Catenulatum Subspecies Present in Infant versus Adult Guts. BMC Microbiol..

[62] Ambrogi V., Bottacini F., O’Sullivan J., O’Connell Motherway M., Linqiu C., Schoemaker B., Schoterman M., van Sinderen D. (2019). Characterization of GH2 and GH42 β-Galactosidases Derived from Bifidobacterial Infant Isolates. AMB Express.

[63] O’Connell K.J., Motherway M.O.C., O’Callaghan J., Fitzgerald G.F., Paul Ross R., Ventura M., Stanton C., van Sinderen D. (2013). Metabolism of Four α-Glycosidic Linkage-Containing Oligosaccharides by Bifidobacterium Breve UCC2003. Appl. Environ. Microbiol..

[64] Nishimoto M., Kitaoka M. (2007). Identification of the Putative Proton Donor Residue of Lacto-N-Biose Phosphorylase (EC 2.4.1.211). Biosci. Biotechnol. Biochem..

[65] Arzamasov A.A., Nakajima A., Sakanaka M., Ojima M.N., Katayama T., Rodionov D.A., Osterman A.L. (2022). Human Milk Oligosaccharide Utilization in Intestinal Bifidobacteria Is Governed by Global Transcriptional Regulator NagR. mSystems.

[66] Odamaki T., Horigome A., Sugahara H., Hashikura N., Minami J., Xiao J.Z., Abe F. (2015). Comparative Genomics Revealed Genetic Diversity and Species/Strain-Level Differences in Carbohydrate Metabolism of Three Probiotic Bifidobacterial Species. Int. J. Genomics.

[67] Garrido D., Ruiz-Moyano S., Jimenez-Espinoza R., Eom H.J., Block D.E., Mills D.A. (2013). Utilization of Galactooligosaccharides by Bifidobacterium Longum Subsp. Infantis Isolates. Food Microbiol..

[68] Katoh T., Ojima M.N., Sakanaka M., Ashida H., Gotoh A., Katayama T. (2020). Enzymatic Adaptation of Bifidobacterium Bifidum to Host Glycans, Viewed from Glycoside Hydrolyases and Carbohydrate-Binding Modules. Microorganisms.

[69] Zabel B., Yde C.C., Roos P., Marcussen J., Jensen H.M., Salli K., Hirvonen J., Ouwehand A.C., Morovic W. (2019). Novel Genes and Metabolite Trends in Bifidobacterium Longum Subsp. Infantis Bi-26 Metabolism of Human Milk Oligosaccharide 2′-Fucosyllactose. Sci. Rep..

[70] Sela D.A., Chapman J., Adeuya A., Kim J.H., Chen F., Whitehead T.R., Lapidus A., Rokhsar D.S., Lebrilla C.B., German J.B. (2008). The Genome Sequence of Bifidobacterium Longum Subsp. Infantis Reveals Adaptations for Milk Utilization within the Infant Microbiome. Proc. Natl. Acad. Sci. USA.

[71] LoCascio R.G., Desai P., Sela D.A., Weimer B., Mills D.A. (2010). Broad Conservation of Milk Utilization Genes in Bifidobacterium Longum Subsp. Infantis as Revealed by Comparative Genomic Hybridization. Appl. Environ. Microbiol..

[72] Sela D.A., Li Y., Lerno L., Wu S., Marcobal A.M., Bruce German J., Chen X., Lebrilla C.B., Mills D.A. (2011). An Infant-Associated Bacterial Commensal Utilizes Breast Milk Sialyloligosaccharides. J. Biol. Chem..

[73] Suzuki R., Wada J., Katayama T., Fushinobu S., Wakagi T., Shoun H., Sugimoto H., Tanaka A., Kumagai H., Ashida H. (2008). Structural and Thermodynamic Analyses of Solute-Binding Protein from Bifidobacterium Longum Specific for Core 1 Disaccharide and Lacto-N-Biose I. J. Biol. Chem..

[74] Barratt M.J., Nuzhat S., Ahsan K., Frese S.A., Arzamasov A.A., Sarker S.A., Munirul Islam M., Palit P., Islam M.R., Hibberd M.C. (2022). Bifidobacterium Infantis Treatment Promotes Weight Gain in Bangladeshi Infants with Severe Acute Malnutrition. Sci. Transl. Med..

[75] Kim T.B., Song S.H., Kang S.C., Oh D.K. (2003). Quantitative Comparison of Lactose and Glucose Utilization in Bifidobacterium Longum Cultures. Biotechnol. Prog..

[76] Parche S., Beleut M., Rezzonico E., Jacobs D., Arigoni F., Titgemeyer F., Jankovic I. (2006). Lactose-over-Glucose Preference in Bifidobacterium Longum NCC2705: GlcP, Encoding a Glucose Transporter, Is Subject to Lactose Repression. J. Bacteriol..

[77] Lanigan N., Kelly E., Arzamasov A.A., Stanton C., Rodionov D.A., van Sinderen D. (2019). Transcriptional Control of Central Carbon Metabolic Flux in Bifidobacteria by Two Functionally Similar, yet Distinct LacI-Type Regulators. Sci. Rep..

[78] Schwab C., Ruscheweyh H.J., Bunesova V., Pham V.T., Beerenwinkel N., Lacroix C. (2017). Trophic Interactions of Infant Bifidobacteria and Eubacterium Hallii during L-Fucose and Fucosyllactose Degradation. Front. Microbiol..

[79] Honda Y., Nishimoto M., Katayama T., Kitaoka M. (2013). Characterization of the Cytosolic β-N-Acetylglucosaminidase from Bifidobacterium Longum Subsp. Longum. J. Appl. Glycosci..

[80] Sakurama H., Kiyohara M., Wada J., Honda Y., Yamaguchi M., Fukiya S., Yokota A., Ashida H., Kumagai H., Kitaoka M. (2013). Lacto-N-Biosidase Encoded by a Novel Gene of Bifidobacterium Longum Subspecies Longum Shows Unique Substrate Specificity and Requires a Designated Chaperone for Its Active Expression. J. Biol. Chem..

[81] Oki K., Akiyama T., Matsuda K., Gawad A., Makino H., Ishikawa E., Oishi K., Kushiro A., Fujimoto J. (2018). Long-Term Colonization Exceeding Six Years from Early Infancy of Bifidobacterium Longum Subsp. Longum in Human Gut. BMC Microbiol..

[82] Yamada C., Gotoh A., Sakanaka M., Hattie M., Stubbs K.A., Katayama-Ikegami A., Hirose J., Kurihara S., Arakawa T., Kitaoka M. (2017). Molecular Insight into Evolution of Symbiosis between Breast-Fed Infants and a Member of the Human Gut Microbiome Bifidobacterium Longum. Cell Chem. Biol..

[83] Sakanaka M., Hansen M.E., Gotoh A., Katoh T., Yoshida K., Odamaki T., Yachi H., Sugiyama Y., Kurihara S., Hirose J. (2019). Evolutionary Adaptation in Fucosyllactose Uptake Systems Supports Bifidobacteria-Infant Symbiosis. Sci. Adv..

[84] Asakuma S., Hatakeyama E., Urashima T., Yoshida E., Katayama T., Yamamoto K., Kumagai H., Ashida H., Hirose J., Kitaoka M. (2011). Physiology of Consumption of Human Milk Oligosaccharides by Infant Gut-Associated Bifidobacteria. J. Biol. Chem..

[85] Gotoh A., Katoh T., Sakanaka M., Ling Y., Yamada C., Asakuma S., Urashima T., Tomabechi Y., Katayama-Ikegami A., Kurihara S. (2018). Sharing of Human Milk Oligosaccharides Degradants within Bifidobacterial Communities in Faecal Cultures Supplemented with Bifidobacterium Bifidum. Sci. Rep..

[86] Turroni F., Strati F., Foroni E., Serafini F., Duranti S., van Sinderen D., Ventura M. (2012). Analysis of Predicted Carbohydrate Transport Systems Encoded by Bifidobacterium Bifidum PRL2010. Appl. Environ. Microbiol..

[87] Hakomori S. (2008). itiroh Structure and Function of Glycosphingolipids and Sphingolipids: Recollections and Future Trends. Biochim. Biophys. Acta Gen. Subj..

[88] Nishimoto M., Kitaoka M. (2007). Identification of N-Acetylhexosamine 1-Kinase in the Complete Lacto-N-Biose I/Galacto-N-Biose Metabolic Pathway in Bifidobacterium Longum. Appl. Environ. Microbiol..

[89] Junick J., Blaut M. (2012). Quantification of Human Fecal Bifidobacterium Species by Use of Quantitative Real-Time PCR Analysis Targeting the GroEL Gene. Appl. Environ. Microbiol..

[90] Wu G., Zhang C., Wu H., Wang R., Shen J., Wang L., Zhao Y., Pang X., Zhang X., Zhao L. (2017). Genomic Microdiversity of Bifidobacterium Pseudocatenulatum Underlying Differential Strain-Level Responses to Dietary Carbohydrate Intervention. MBio.

[91] Matsuki T., Watanabe K., Fujimoto J., Kado Y., Takada T., Matsumoto K., Tanaka R. (2004). Quantitative PCR with 16S RRNA-Gene-Targeted Species-Specific Primers for Analysis of Human Intestinal Bifidobacteria. Appl. Environ. Microbiol..

[92] Turroni F., Peano C., Pass D.A., Foroni E., Severgnini M., Claesson M.J., Kerr C., Hourihane J., Murray D., Fuligni F. (2012). Diversity of Bifidobacteria within the Infant Gut Microbiota. PLoS ONE.

[93] Kiyohara M., Tachizawa A., Nishimoto M., Kitaoka M., Ashida H., Yamamot K. (2009). Prebiotic Effect of Lacto-n-Biose i on Bifidobacterial Growth. Biosci. Biotechnol. Biochem..

