# Supplementary figures and images for: Variation in the Conservation of Species-Specific Gene Sets for HMO Degradation and Its Effects on HMO Utilization in Bifidobacteria
Source: Nutrients. 2024 Jun 15;16(12):1893. doi: 10.3390/nu16121893 (PMC11206791; doi:10.3390/nu16121893)

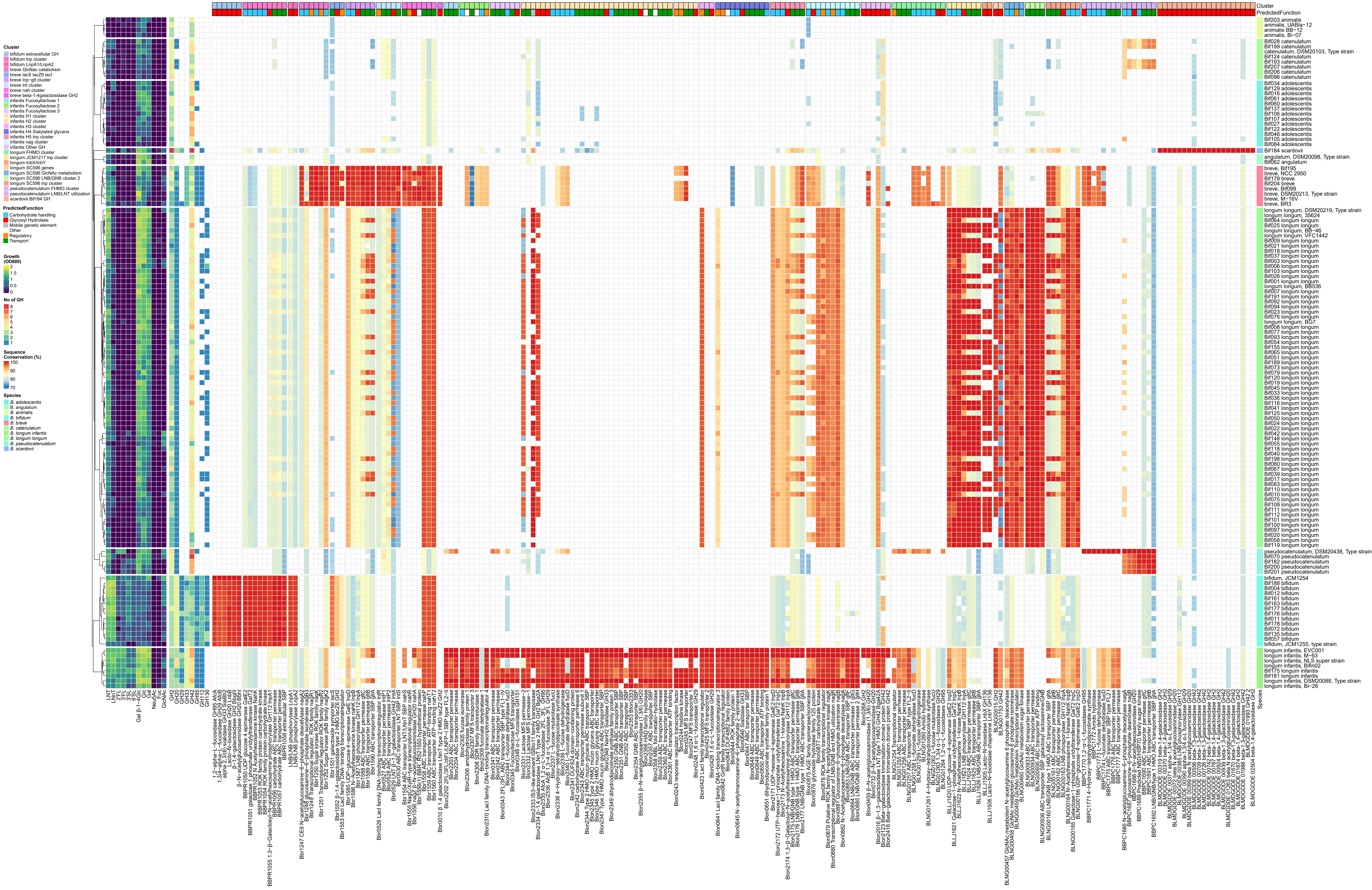

Supplement: Supplementary file 1 [file nutrients-16-01893-s001.zip › Supplementary figure S1. Complete Heatmap FINAL.pdf]

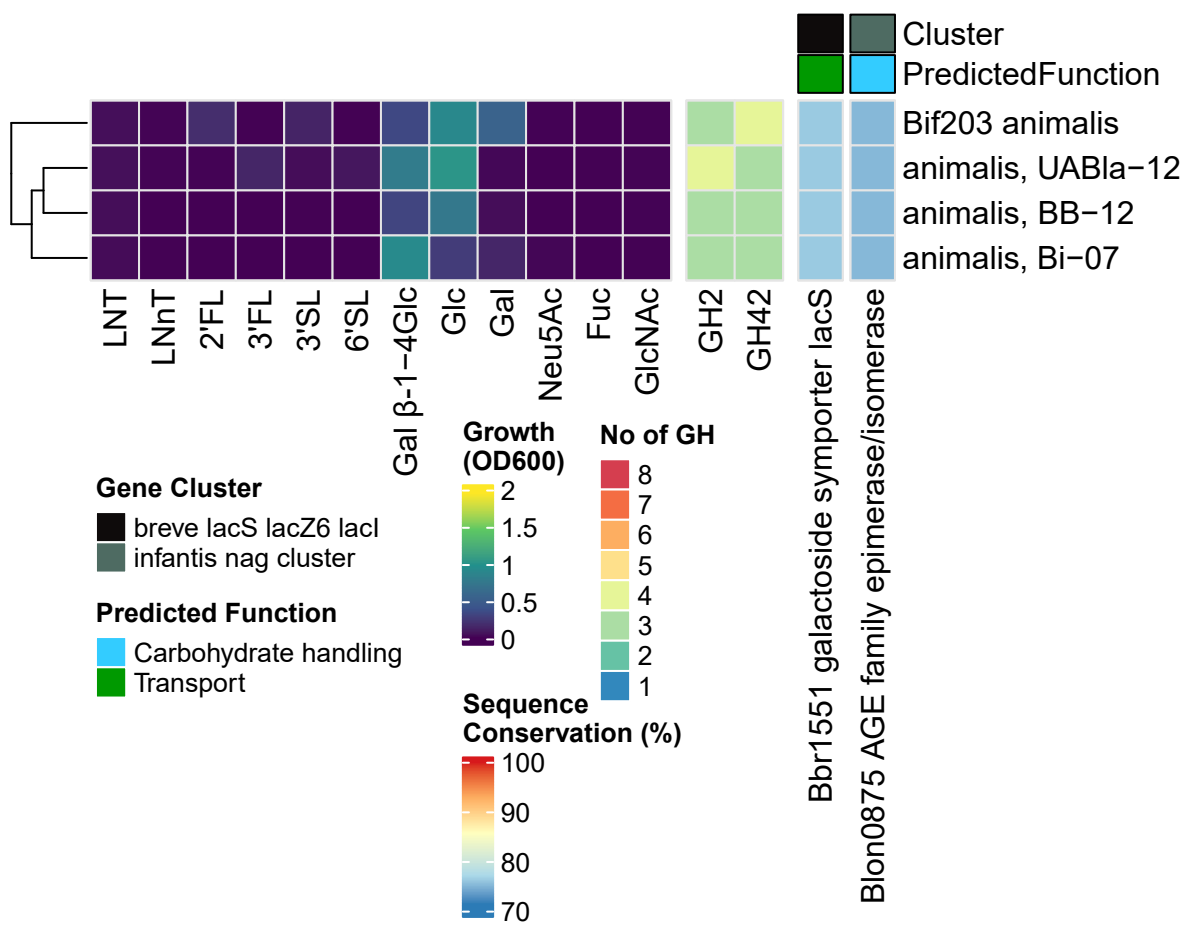

Supplement: Supplementary file 1 [file nutrients-16-01893-s001.zip › Supplementary figure S2. heatmap animalis FINAL.pdf]

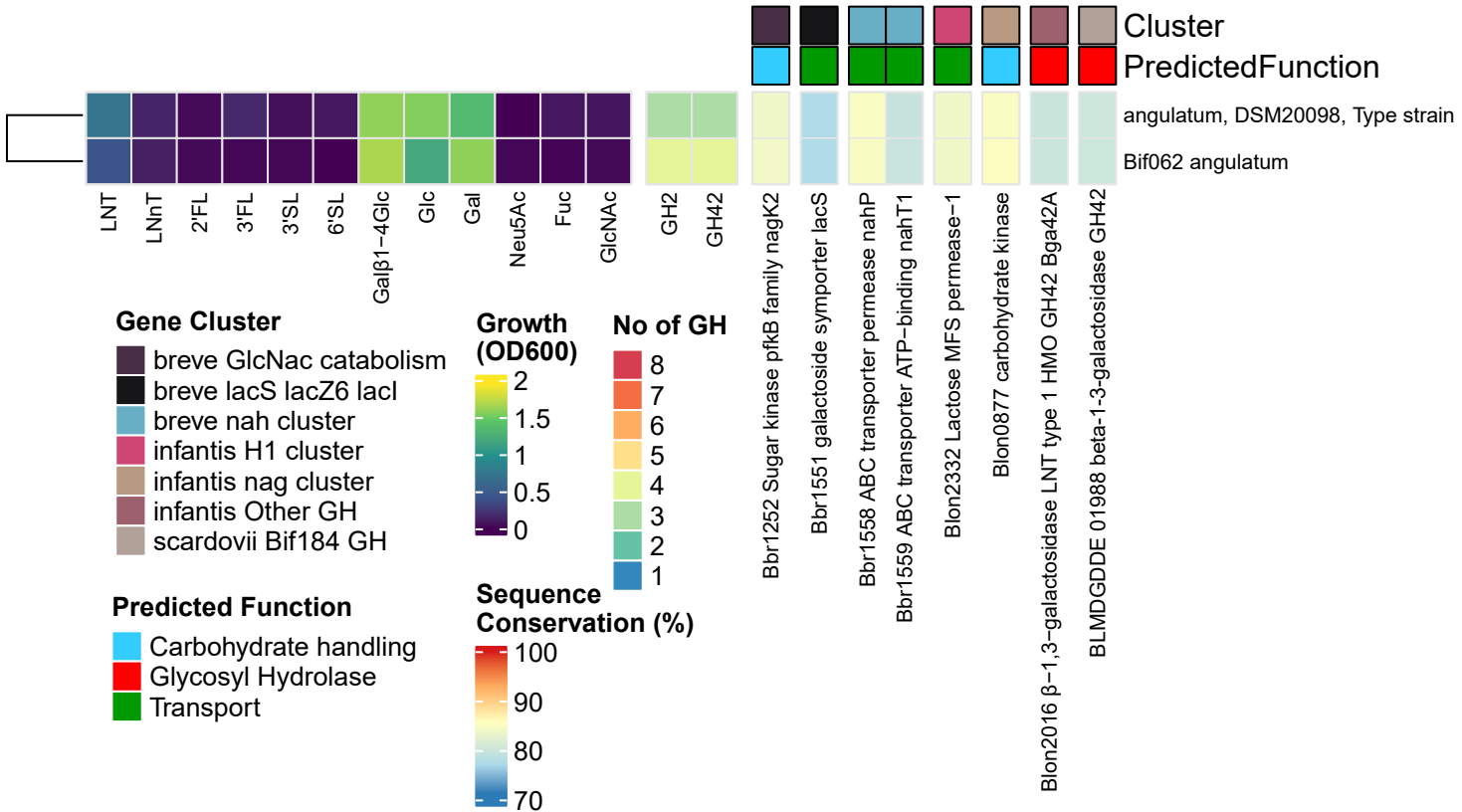

Supplement: Supplementary file 1 [file nutrients-16-01893-s001.zip › Supplementary figure S3. heatmap angulatum FINAL.pdf]
